# Changes in self-reported cannabis use during the COVID-19 pandemic: a scoping review

**DOI:** 10.1186/s12889-023-17068-7

**Published:** 2023-11-01

**Authors:** Kamna Mehra, Jennifer Rup, Jessica L. Wiese, Tara Marie Watson, Sarah Bonato, Sergio Rueda

**Affiliations:** 1 Institute for Mental Health Policy Research, Centre for Addiction and Mental Health, 33 Ursula Franklin Street, Toronto, ON M5S 2S1 Canada; 2 Campbell Family Mental Health Research Institute, Centre for Addiction and Mental Health, 250 College Street, Toronto, ON M5T 1R8 Canada; 3 Department of Family and Community Medicine, Temerty Faculty of Medicine, University of Toronto, 500 University Avenue, Toronto, ON M5G 1V7 Canada; 4 Library Services, Centre for Addiction and Mental Health, 33 Ursula Franklin Street, Toronto, ON M5S 2S1 Canada; 5 Department of Psychiatry, Temerty Faculty of Medicine, University of Toronto, 250 College Street, Toronto, ON M5T 1R8 Canada; 6 Institute of Health Policy, Management and Evaluation, Temerty Faculty of Medicine, University of Toronto, 1 King’s College Circle, Toronto, ON M5S 1A1 Canada

**Keywords:** Cannabis, COVID-19, Scoping review

## Abstract

**Background:**

The COVID-19 pandemic is affecting mental health and substance use (MHSU) issues worldwide. The purpose of this study was to characterize the literature on changes in cannabis use during the pandemic and the factors associated with such changes.

**Methods:**

We conducted a scoping review by searching peer-reviewed databases and grey literature from January 2020 to May 2022 using the Arksey and O’Malley Framework. Two independent reviewers screened a total of 4235 documents. We extracted data from 129 documents onto a data extraction form and collated results using content analytical techniques.

**Results:**

Nearly half (48%) of the studies reported an increase/initiation of cannabis use, while 36% studies reported no change, and 16% reported a decrease/cessation of cannabis use during the pandemic. Factors associated with increased cannabis use included socio-demographic factors (e.g., younger age), health related factors (e.g., increased symptom burden), MHSU factors (e.g., anxiety, depression), pandemic-specific reactions (e.g., stress, boredom, social isolation), cannabis-related factors (e.g., dependence), and policy-related factors (e.g., legalization of medical/recreational cannabis).

**Conclusion:**

Public health emergencies like the COVID-19 pandemic have the potential to significantly impact cannabis use. The pandemic has placed urgency on improving coping mechanisms and supports that help populations adapt to major and sudden life changes. To better prepare health care systems for future pandemics, wide-reaching education on how pandemic-related change impacts cannabis use is needed.

**Supplementary Information:**

The online version contains supplementary material available at 10.1186/s12889-023-17068-7.

## Introduction

The COVID-19 pandemic is affecting the health of populations worldwide, including those living with mental health and substance use concerns [1–3]. Public health measures designed to protect public health by reducing person-to-person contact and virus transmission (e.g., physical distancing rules, closures of in-person offices, businesses, and educational institutions, and cancellation of public gatherings and events) are linked to increases in social isolation, worsening mental health symptoms, and drug-related harms [4–6].

Emerging evidence shows that the COVID-19 pandemic has influenced patterns of cannabis consumption; some studies have reported increases [7–9] or decreases in cannabis use [10, 11], while others have reported on the proportion of the population that increased, decreased, and did not change cannabis use during the pandemic [12–16]. Factors associated with changes in cannabis use during the pandemic include socio-demographic factors (e.g., age, sex, and gender), social isolation, boredom, depression, and anxiety about the pandemic [5, 6, 9, 17].

Chong et al. (2022) [18] conducted a scoping review of academic and grey literature on cannabis use from the onset of the COVID-19 pandemic to February 2021. In this review, the authors conducted a thematic analysis and identified themes related to cannabis use trends, context of use, modes of consumption, and factors contributing to and inhibiting use. This review showed that 33 out of the 76 documents examined change in cannabis use during the first year of the pandemic; of these, one-third reported increased cannabis use during the early phase of the pandemic. It also identified factors contributing to increased cannabis use, including boredom, anxiety, depression and accessibility to cannabis. In our scoping review, we explicitly focus on changes in cannabis use and expand the time frame to cover the first 2.5 years of the pandemic. We also report on a wider range of factors associated with such changes (e.g., socio-demographic, health-related, and policy-related factors). Our scoping review updates the literature and offers a new resource to those studying the effects of the pandemic on cannabis use and the factors that influence changes in patterns of use.

Our aim was to characterize the literature pertaining to changes in cannabis use during the COVD-19 pandemic. The objectives were to: (1) examine how the COVID-19 pandemic affected cannabis use among adults, and (2) to determine what factors were associated with changes in cannabis use during the COVID-19 pandemic.

## Methods

The methodology for this scoping review was based on established guidelines by Arksey and O’Malley (2005) [19], supplemented by Levac et al. (2010) [20]. We identified research questions aligned with the study objectives to guide the scoping review, identified relevant literature through academic and grey literature searches, selected studies based on pre-determined inclusion and exclusion criteria, conducted data extraction using a team based approach, and conducted a descriptive analysis to synthesize and report the data. We implemented a rigorous and standardized team-based review process that adheres to the Preferred Reporting Items for Systematic Review and Meta-Analysis (PRISMA) reporting standards [21] (Supplementary Material 1). We did not provide a critical assessment of the quality of the evidence as this is an emerging area of research.

### **Step 1: identifying the research questions**

We set out to answer the following two sets of questions: (1) How has the COVID-19 pandemic impacted cannabis use among adults? Has cannabis use increased, not changed, or decreased during the pandemic? In addition, have there been changes in types of cannabis consumption (e.g., recreational, medical) or modes of consumption (e.g., smoking, vaping)? (2) What factors were associated with changes in cannabis use during the COVID-19 pandemic? What are the socio-demographic factors, mental health factors, other substance use factors, pandemic related factors (e.g., COVID-19 related regulations, self-isolation), and other factors (e.g., accessibility, legalization) associated with changes in cannabis use during the pandemic?

### Step 2: identifying relevant studies

We developed the search strategies in collaboration with a health science librarian (SB) to identify relevant published peer-reviewed literature and grey literature. For the peer-reviewed literature, we searched the bibliographic databases MEDLINE, Embase, PsycInfo, Scopus, Web of Science, LitCovid, and WHO COVID-19 using both subject headings (e.g., MeSH headings) and keywords to identify relevant research on cannabis and COVID-19. The search results were limited to studies published from January 1, 2020 to May 27, 2022, without any regional, gender or language restrictions. The reference lists of relevant studies were also checked to identify additional studies. The MEDLINE search strategy and a list of sites searched are available in Supplementary Material 2.

The grey literature search was international in scope and utilized a multipronged search approach to identify relevant research. The aims of the grey literature search were to locate information produced by either governmental agencies (e.g., Statistics Canada) or not-for-profit organizations (e.g., Canadian Centre on Substance Abuse), as well as unpublished research available from preprints. To identify relevant publications from governmental agencies and also non-governmental organizations, the search methods utilized grey literature search checklists [22, 23], a Google search using advanced search commands and a supplementary search in the WHO COVID-19 database. In addition, OSF preprints, medRxiv, and bioRxiv were searched for preprints. The grey literature search was also limited to records published since January 1, 2020.

We included both academic literature and grey literature articles published in 2020 or later that met the eligibility criteria for the scoping review. This included peer-reviewed articles utilizing diverse methodologies (e.g., surveys, interviews, randomized controlled trials) and documents/reports/publications from governments/health authorities and non-governmental organizations available online that reported changes in cannabis use during the COVID-19 pandemic among people aged 18 years or older. Eligibility criteria were developed based on team discussions. Publications and other sources were excluded if they: (1) did not include information about changes in cannabis use during the COVID-19 pandemic; (2) only included information about children/youth less than 18 years old; (3) had been published in a language other than English; (4) were duplicate documents; (5) were case reports, letters to the editor, commentaries, viewpoints, corrigendum, or conference abstracts/proceedings; (6) included cannabis use as a predictor but not the outcome of interest; (7) were about cannabis as a treatment option for COVID-19 infection; (8) describe cannabis use that was not self-reported (e.g., waste-water measurements, anonymous location data measurements, emergency department visits,); (9) were about the effects of cannabis on health issues during the pandemic (e.g., effect of cannabis use on COVID-19 infection, EVALI, or other health issues); and (10) were reviews or protocols for reviews.

### Step 3: study selection

**Fig. 1:**
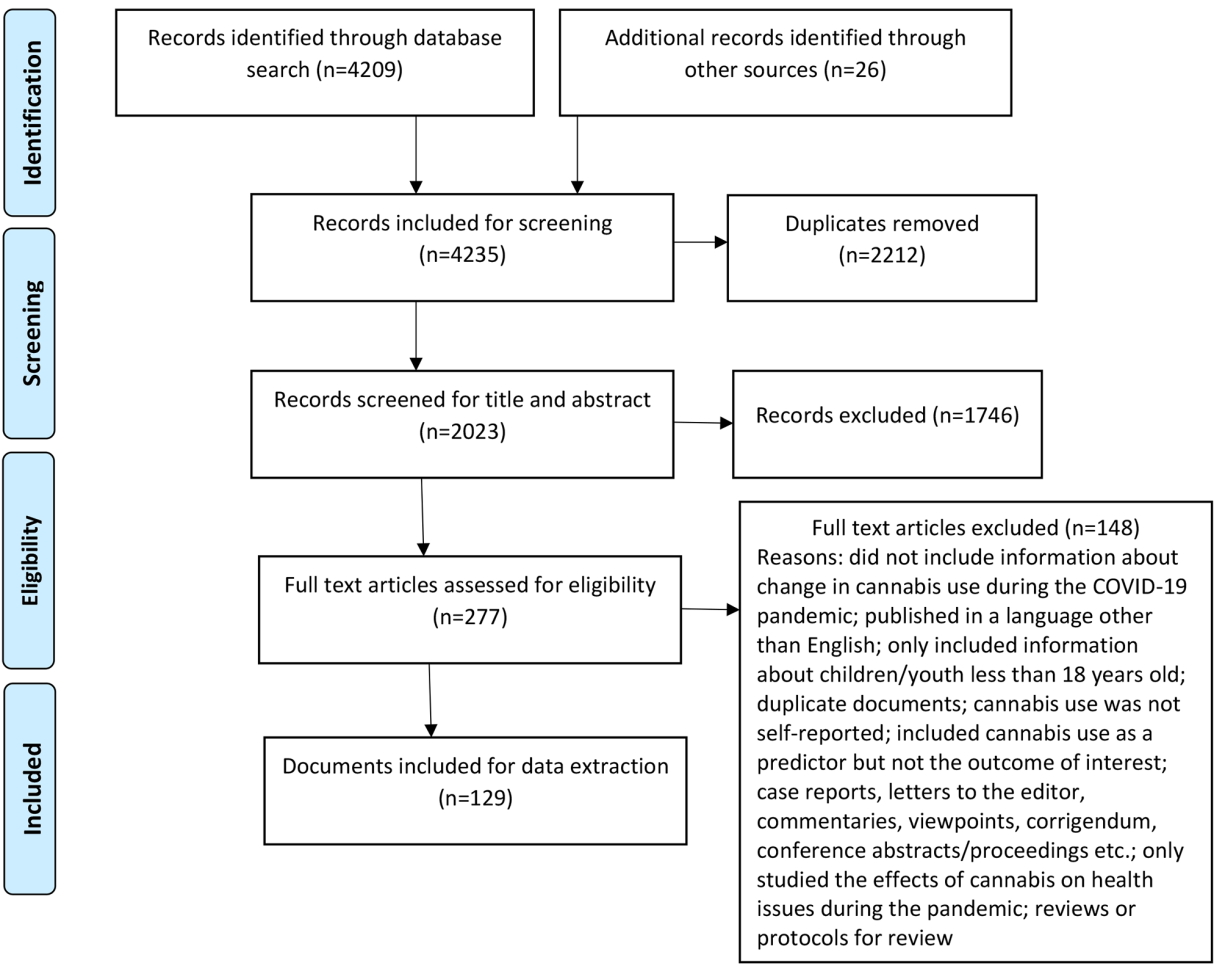
Flowchart of documents included, based on PRISMA guidelines

We used the Covidence online software program for systematic reviews [24] for the review process. A total of 4209 peer reviewed documents (MEDLINE n = 461, Embase n = 716, PsychInfo n = 104, Scopus n = 1108, Web of Science n = 576, LitCovid n = 280, WHO COVID database n = 766, medRxiv and bioRxiv n = 183, OSF preprints n = 15), 23 grey literature documents, and 3 additional references (i.e., two peer reviewed and one grey literature) were included for screening (Fig. 1). At title and abstract review stage, 2212 duplicate documents were removed. KM and JR independently screened 2023 titles and abstracts of the identified literature to determine eligibility based on established criteria. Conflicts were resolved through discussion with a third reviewer (SR). Following the title and abstract review stage, 277 documents were identified as eligible for full text review. These full text documents were independently reviewed by two reviewers (JR and KM) and any conflicts were resolved through discussion with a third reviewer (SR). A total of 129 documents (116 peer-reviewed articles, 13 grey literature documents) were included for data extraction.

### Step 4: charting the data

To chart the data, the team developed, piloted and modified a tool detailing instructions and formatting guidelines for data extraction. KM and JR utilized this tool to independently extract information from approximately half the documents each using a Microsoft Excel [25] form and reviewed each other’s data extraction to ensure consistency. The following information was extracted: publication information (e.g., authors, publishing year), study location, legal status of cannabis where study was conducted, level of pandemic-related public health measures where study was conducted, study design, methodology, method, sample information and demographics, type of cannabis use (e.g., medical or recreational), modes of cannabis consumption (e.g., smoking, vaping, edibles), outcome measures, tool(s) used for measurement of change in cannabis use and associated factors, qualitative findings, and author’s main results and conclusion/interpretation for changes in cannabis use and/or associated factors.

### Step 5: collating, summarizing and reporting the results

The information collected using the data extraction tool was quantitatively and descriptively summarized. In the Supplementary Material 3, Table A provides characteristics of the documents included in the review and Table B synthesizes results from our descriptive analysis of textual data. We met weekly to discuss progress, reporting, and interpretations. To effectively organize the data, the documents were broadly classified into population groups and then sub-grouped based on the change in cannabis use. For changes in cannabis use, studies were categorized as demonstrating *increased use* when reporting a statistically significant increase in use, a higher proportion of participants reporting increased use compared to no change or decreased use, or only reporting increased use; studies were categorized as *no change* when reporting no significant change in cannabis use, a higher proportion of participants reporting no change than increased or decreased use, or only reporting no change in cannabis use; and studies were categorized as *decreased use* when reporting significant decrease in cannabis use, a higher proportion of participants reporting decreased than no change or increased use, or only reporting decreased cannabis use during the pandemic. Qualitative data were similarly organized as increase, no change, or decrease, based on how many participants reported the direction of change in cannabis use. Among studies that reported diverse changes in prevalence, frequency, and/or quantity of cannabis use, the change in prevalence of cannabis use was prioritized over change in frequency, which was in turn prioritized over change in quantity of cannabis. Since there was high variability in the analysis conducted by the individual studies, with some adjusting for different sets of covariates and some not at all, we reported a general statistical change in cannabis use, i.e., increase, decrease, or no change, without specifying the covariates.

Only those factors that were associated with changes in cannabis use during the pandemic were included in this review. Factors associated with change in cannabis use from qualitative studies were extracted based on the participants’ descriptions (e.g., if participants mentioned that boredom during the pandemic increased their cannabis use). When factors were similar in nature, e.g., social isolation/loneliness/reduced social connection, they were organized into one category and then used to compare change in cannabis use. Additionally, there were mixed associations among factors associated with change in cannabis use across studies. For example, there were some instances where one study found a certain factor associated with increase in cannabis use and another study found the same factor associated with decrease in cannabis use (e.g., financial concerns associated with decrease in cannabis use in Salles et al. (2021) [10] and with increase in cannabis use in Imtiaz et al. (2021) [26]). All factors associated with cannabis use have been detailed by study in the Supplementary Material 3 (Table B), but for ease of interpretation only those factors that were consistently associated with either increase, decrease, or no change in cannabis use across studies were described in the results.

## Results

### Characteristics of the included studies

Table A in Supplementary Material 3 describes the characteristics of included studies. Out of the 129 documents included in this scoping review, studies were most commonly conducted in the USA (n = 50, 39%) or Canada (n = 28, 22%). Most of the included studies utilized cross-sectional (n = 82, 64%), longitudinal (n = 21, 16%), or repeated cross sectional (n = 14, 11%) study designs. Data collection was most commonly conducted through online surveys (n = 109, 85%), whereas a few studies collected data through in person/online/phone surveys (n = 10, 8%), in person/online/phone interviews (n = 8, 6%), or mixed surveys and interviews (n = 3, 2%). The sample sizes ranged from 16 to 227,258, ages ranged from a mean of 15.3 years to 58.1 years, proportion of females ranged from 9 to 88%, and proportion of Caucasian participants ranged from 11 to 92%.

The seven population groups included: (1) general adult population (n = 33, 26%); (2) young adults/students (n = 41, 32%); (3) people who use cannabis (n = 14, 11%); (4) people with health/mental health comorbidities (n = 10, 8%); (5) people who use substances (n = 17, 13%); (6) occupational groups (e.g., healthcare workers, veterans, athletes) (n = 7, 5%); and (7) sexual minority/2SLGBTQIA + groups (n = 7, 5%) (Table 1). Three articles had an overlap between two population groups (i.e., young adults/students as well as people who use cannabis, sexual minority/2SLGBTQIA + groups as well as people who use cannabis, and people with health/mental health comorbidities as well as people who use cannabis) [27–29], but since they all included information about people who use cannabis, these were included in that specific group.

Out of the 129 studies, only seven studies specifically examined change in type of cannabis use (i.e., medical or recreational cannabis use) [17, 29–34]. However, several studies assessed changes in specific modes of cannabis consumption [5, 8, 30, 32, 33, 35–45]. In addition, several studies described the level of public health measures during the COVID-19 pandemic (e.g., stay-at-home orders, ban on gatherings, closure of non-essential businesses and schools) (Supplementary Material 3 - Table A). Of particular note, there were three studies that mentioned that cannabis dispensaries were deemed essential businesses and remained open during the pandemic [7, 30, 46]. Furthermore, three studies described different levels of public health measures at different stages of the pandemic and changes in cannabis use based on these measures [17, 47, 48]. Overall, several studies mentioned the legal status of cannabis in the area where the study was conducted: 16 studies (12%) mentioned legal/decriminalized status, 27 studies (21%) mentioned illegal status, and 86 studies (67%) were conducted in areas with both legal and illegal status (variable status) or did not specify the legal status of cannabis; however, only five studies statistically examined the changes in cannabis use during the pandemic based on the legal status of cannabis [4, 7, 30, 33, 49].

### Change in cannabis use during the COVID-19 pandemic

#### Change in frequency or quantity of cannabis use

Table 1 and Table B in Supplementary Material 3 describes changes in cannabis use during the COVID-19 pandemic and the factors associated with those changes. Out of the 129 studies included in this scoping review, 63 (48%) reported increasing/initiating cannabis use, 46 (36%) reported no change, and 20 (16%) reported decreasing/cessation of cannabis use. Of particular note, 12 studies reported 0.3–17% of participants initiated cannabis use [8, 10, 11, 40, 43, 45, 50–55] and 14 studies found 0.7–65% of participants reported cessation of cannabis use during the COVID-19 pandemic [8, 10, 11, 16, 32, 39–41, 43, 45, 50, 53, 55, 56]. Table 1 specifies the change in cannabis use among different population categories, organized by increase/initiation, no change, or decrease/cessation in cannabis use within each of these populations.

#### Change in cannabis use among different population groups

**Fig. 2:**
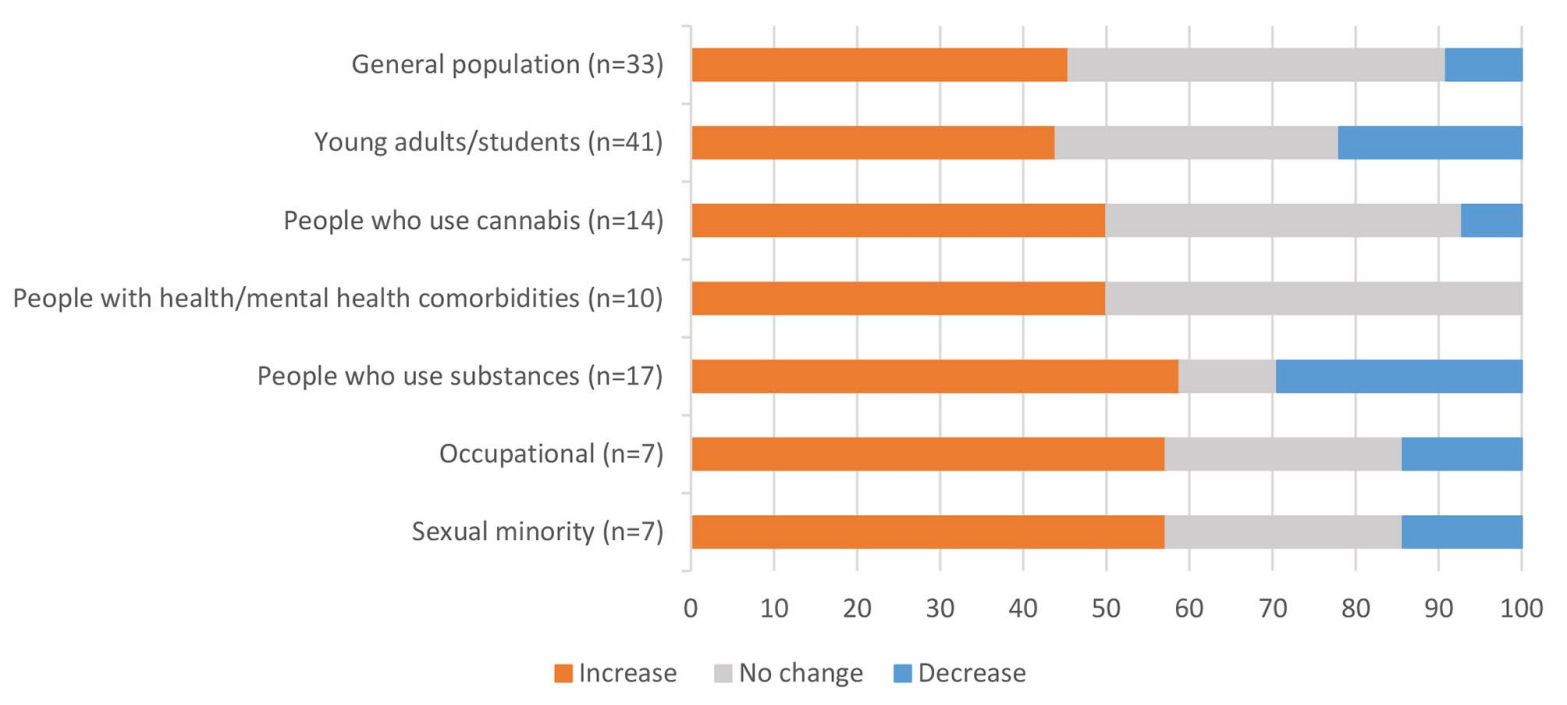
Percentage of documents with each population group reporting change in cannabis use during the COVID-19 pandemic

The young adult/student population category contained the highest number of studies (n = 41), and a majority of studies reported an increase in cannabis use during the pandemic (n = 18, 44%), compared to no change (n = 14, 34%), or a decrease (n = 9, 22%) in cannabis use. Out of the 33 studies conducted among the general adult population, 15 (46%) reported an increase, 15 (46%) reported no change, and 3 (9%) reported a decrease in cannabis use. Among studies about people who use substances (n = 17), the majority found an increase in cannabis use during the pandemic (n = 10, 59%), whereas a few found a decrease (n = 5, 29%) or no change (n = 2, 12%) in cannabis use. Half the studies conducted among people who use cannabis (n = 7, 50%) found an increase in cannabis use, whereas 6 (43%) found no change and only one found a decrease (7%) in cannabis use. Half the studies conducted among people with health/mental health comorbidities (n = 5, 50%) found an increase in cannabis use and half (n = 5, 50%) found no change in cannabis use, with no study finding a decrease in cannabis use. Out of the seven studies conducted in occupational as well as sexual/gender minority groups, four (57%) found an increase, two (29%) found no change and one (14%) found a decrease in cannabis use (Table 1; Fig. 2).

Thus, a majority of studies in most of the studied populations reported an increase in cannabis use as compared to decrease or no change during the COVID-19 pandemic. These findings may differ based on how cannabis use was measured in each of these studies and which measurement was prioritized during our categorization of data. In addition, very few studies included representative population data or utilized similar methodologies or time frames for data collection.

#### Change in type of cannabis use and modes of administration

Studies measuring change in the type of cannabis use reported mixed results, that is, two studies found an increase in recreational cannabis use [17, 31] and one found a decrease [34]; two studies found no change in medical cannabis use [29, 30] and two found an increase in use [32, 33]. Similarly, studies measuring change in smoking as a mode of cannabis consumption reported variable results, for example, three studies found an increase in smoking [5, 32, 41], two found a decrease [8, 43], and three found no change [40, 42, 45]. However, more studies found an increase in other modes of consumption rather than no change or decrease. For example, five studies found an increase in vaping [5, 36, 37, 43, 44], and three found no change [39, 42, 45], whereas four studies found an increase in the use of edibles [5, 30, 35, 43], one found no change [45] and one found a decrease [38]. Thus, data about the change in types and modes of cannabis consumption revealed variable findings. However, there was a lack of standardized measurements while studying such changes, for example, the most common method of data collection was a survey question about change in cannabis use with the options of increase, no change, or decrease, and it was only used among 28 (22%) of documents (Appendix C).

### Factors associated with change in cannabis use

#### Socio-demographic factors

Among the studies conducted with the general adult population (age range 15 to 75 + years), younger people were found to increase their cannabis use [12, 14, 16, 26, 57–61]. However, among studies conducted with young adults/students (age range 15 to 29 years), older members of these younger groups were found to increase cannabis use [62, 63]. In general, more studies reported increased cannabis use among females [7, 12, 30, 32, 33, 39, 41] than males [46, 59, 64]. Those who identified as a sexual/gender minority [28, 65, 66], had lower income [7, 59], lower educational levels [16, 26, 53, 60], or were unemployed or lost employment [27, 32, 67, 68], were found to be more likely to increase/initiate cannabis use during the pandemic. Two studies found increased cannabis use among non-Caucasians [27, 63] whereas one study found increased use among Caucasians [7]. In addition, those living alone [53, 69], or not living with parents [64, 68, 70] were found to increase cannabis use and those living with children [53] or with parents [38, 70] were found to decrease cannabis use.

#### Health-related factors

A few studies examined the impact of the pandemic on cannabis use in relation to concerns about general health, or physical health disorders such as endometriosis or obesity. For example, those who were focused on their health during the pandemic were found to decrease their cannabis use [71, 72], whereas those who experienced health issues (e.g., obesity) [16, 73], increased symptom burden [4, 35], or reduced access to healthcare [35] were found to increase/initiate cannabis use during the pandemic.

#### Mental health or other substance use factors

Those who experienced anxiety [5, 6, 12, 13, 29, 35, 42, 43, 45, 57, 74–77], depression [5, 6, 12, 13, 29, 43, 45, 57, 62, 70, 71, 74, 75], PTSD [55, 65], suicidal ideation [78], impulsivity [65], and lower dispositional resilience [68] were found to increase cannabis use. People who experienced polysubstance/use of substances other than cannabis [4, 47, 58] or substance use concerns [79] were found to increase cannabis use during the pandemic.

#### Pandemic specific reactions

Those who experienced stress [5, 8, 12, 35, 42, 58, 61, 72, 75, 80, 81], boredom [4, 10, 36, 38, 41, 43, 45, 54, 61, 71, 77, 80, 81], more free time [33, 38, 42, 43, 45, 71], disrupted daily routine [42, 80, 81], work from home [32, 72], or social isolation/reduced social support [5, 8, 12, 17, 47, 54, 61, 74, 80, 82] due to the pandemic were found to increase/initiate cannabis use. Those who perceived that cannabis use would not increase their risk of COVID-19 [55] or those who experienced COVID-19 symptoms [32] increased cannabis use, whereas those who were at risk of COVID-19 [33] or perceived more harm due to COVID-19 [42] decreased their cannabis use. Changes in cannabis use based on the level of public health measures revealed mixed results, with one study reporting decreased cannabis use with easing of lockdown measures [17] and two reporting increased cannabis use with easing of/lesser lockdown measures [47, 48].

#### Cannabis-related factors

Those who experienced cannabis craving/dependence [8, 39, 83], had easier access to cannabis [8, 61, 81], or had higher stockpile of cannabis [80] were found to increase/initiate cannabis use whereas decrease/cessation of cannabis use was reported among those who had reduced opportunities to consume cannabis [38, 41, 42, 61, 71, 77, 80, 84], reduced access to cannabis [4, 35, 36, 77, 84], or lower stockpile of cannabis [72, 80].

#### Policy-related factors

Among studies conducted with the general adult population/people who use cannabis, people who lived in areas where medical and recreational cannabis use was legal [30, 33, 81], or only medical cannabis use was legal [7], were found to increase/initiate cannabis use, whereas those who lived in areas where cannabis was prohibited/illegal were found to decrease cannabis use [4, 7] during the pandemic. One study conducted by Graupensperger et al. (2021) [49] among young adults found no change in cannabis use among participants who lived in Washington, where cannabis was legal, compared to those who lived outside Washington.

**Fig. 3:**
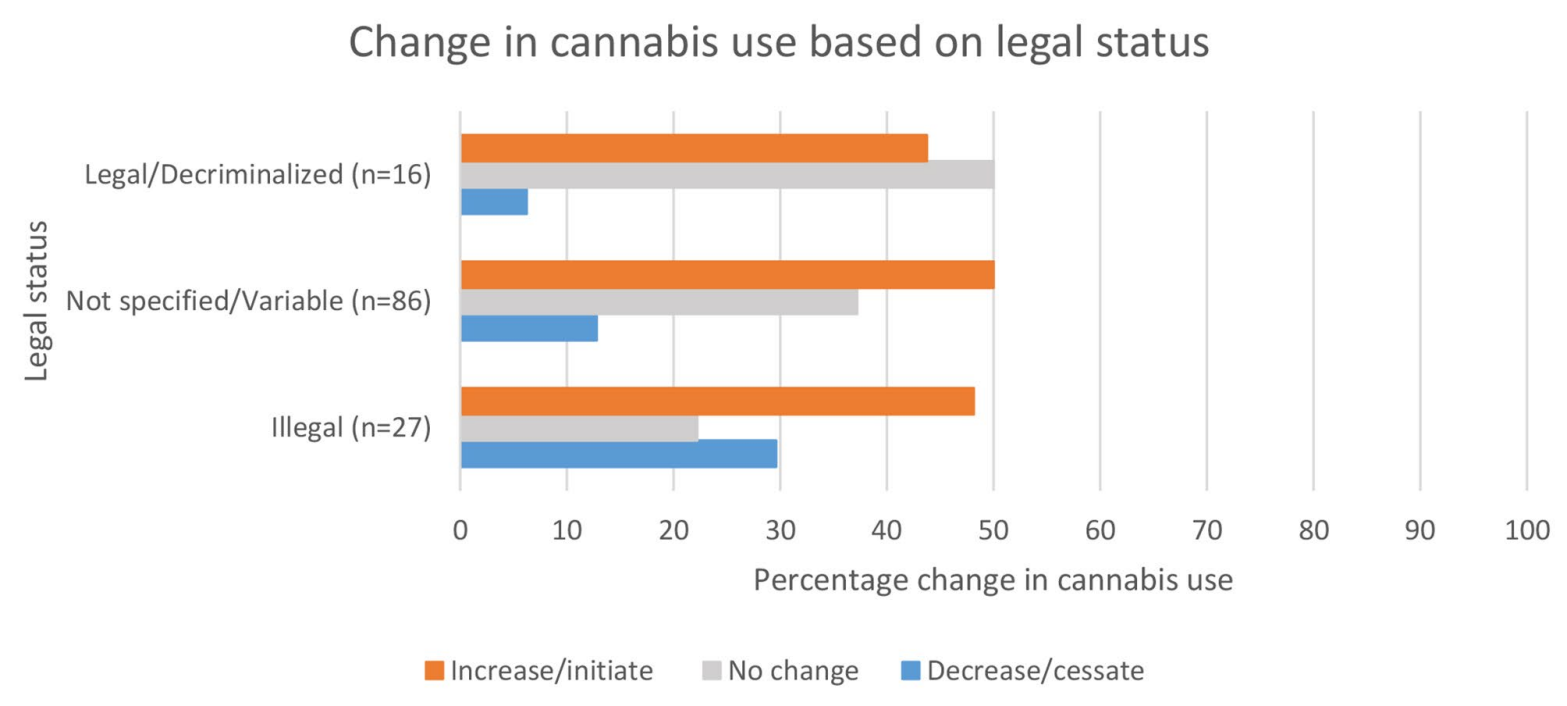
Percentage of documents reporting change in cannabis use during the COVID-19 pandemic based on the legal status of cannabis where study was conducted

Regardless of whether studies assessed change in cannabis use based on cannabis legal status, out of the studies that were conducted in legal/decriminalized jurisdictions, 7 (44%) reported increase, 8 (50%) reported no change, and 1 (6%) reported decrease in cannabis use; among studies conducted in jurisdictions were cannabis was illegal, 13 (48%) reported increase, 6 (22%) reported no change, and 8 (30%) reported decrease in cannabis use; and studies that did not specify legal status or had variable legal status (conducted in area with both legal or illegal status), 43 (50%) reported increase, 32 (37%) reported no change, and 11 (13%) reported decrease in cannabis use. Figure 3 shows a step-wise increase in the proportion of studies that document decreases or cessation of cannabis use going from legal to illegal jurisdictions.

## Discussion

Our scoping review examined how the COVID-19 pandemic impacted cannabis use and reported on factors associated with these changes. More studies were found to report increased cannabis use during the pandemic among youth/students, people who use cannabis, people who use other substances, occupational groups, and sexual/gender minority groups than no change or decrease in cannabis use during the pandemic. These patterns seem to be consistent with existing evidence indicating that greater cannabis use is more prevalent among specific populations (e.g., youth, people who use substances, and sexual minorities) compared to the general population [85–87]. An equal number of studies reported no change or increased cannabis use among the general population and people with health/mental health comorbidities, and among studies reporting decreases in cannabis use, a majority of studies belonged to the young adult/student group. The finding about initiation and cessation of cannabis use suggests that the pandemic may have acted as a trigger for starting as well as stopping cannabis use. Our scoping review shows a higher proportion of studies reporting an increase/initiation in cannabis use (48%) compared to a recent review conducted by Chong et al. (2022) [18] in which 11 out of 33 studies (33.3%) found more users reporting increased cannabis use than decreased, and 27% of studies reporting stable use. In addition, the review by Chong et al. (2022) [18] did not include studies examining initiating cannabis use during the COVID-19 pandemic whereas this scoping review identified several studies reporting on initiation and cessation of cannabis use during the pandemic. The divergent findings between the two scoping reviews could be due to a number of factors including the longer observation period in this scoping review (2.5 years vs. 1 year), narrower inclusion criteria focusing on changes in cannabis use, and more specific criteria for the categorization of change in cannabis use utilized in this scoping review.

Several socio-demographic factors were studied in relation to changes in cannabis use. Some studies found younger age to be associated with increases in cannabis use during the pandemic. Data about the factors associated with increases in cannabis use among young adults/students revealed factors similar to the adult population, such as experiencing mental health concerns, boredom, social isolation, and stress during the pandemic. However, the review found mixed reports about changes in cannabis use with respect to gender and ethnicity. Some findings in this review such as increased cannabis use among lower income and lower education groups may illustrate the need for healthcare decision makers to develop strategies that focus on the healthcare needs of specific population groups [12, 27].

Two studies reported that cannabis inhalation reduced during the COVID-19 pandemic due to concerns related to respiratory health [30, 35], however, other studies examining changes in smoking or vaping found variable results [5, 36, 37]. Similarly, studies examining changes in recreational or medical cannabis use due to the COVID-19 pandemic reported variable results [17, 29–34]. The diverse population groups and methodologies utilized to assess change in modes and types of cannabis use could potentially contribute to inconsistent findings. There is a need to standardize the measurement of types of cannabis consumption and modes of consumption (i.e., examining changes in smoking, vaping, edibles), as the lack of consensus on how cannabis should be measured limits the ability to accurately assess cannabis use and generalize its effects [88]. Further research is needed to understand the impact that health emergencies such as pandemics may have on consumption of different modes or types of cannabis use, including changes in frequency of use and the quantity consumed.

The specific pandemic related factors found to be related to increases in cannabis use (e.g., social isolation and disrupted daily routine) as well as mental health factors (e.g., anxiety and depression) point to a need for additional public health supports and strategies for managing the stressors and mental health impacts associated with the pandemic [4, 5, 34, 62, 81]. In addition, factors specifically associated with initiating cannabis use during the pandemic included amplification of health issues, reduced access to health care, and mental health impacts of the pandemic. Findings suggest a need to maintain access to health care and other supports during pandemics. Strategies to improve access to an already overburdened healthcare system include utilizing digital health applications such as telehealth approaches, modifying treatment plans to include newer technological approaches, and providing options for in-person versus remote care [89, 90].

The mixed findings about changes in cannabis use associated with public health measures (e.g., lockdowns) could be attributed to other associated factors. For example, increased stress due to stricter lockdowns could be related to increased cannabis use, and increased access and increased social opportunities to consume cannabis during easing of lockdown measures could be related to increased cannabis use. Three studies mentioned cannabis dispensaries were deemed essential and found increased cannabis use, however, this public health measure was not studied as a factor associated with change in cannabis use in these studies. In addition, the studies that assessed changes in cannabis use in the general population associated with legalization found increased cannabis use in jurisdictions where recreational and/or medical cannabis was legal. Similarly, most studies that were conducted in legal/decriminalized jurisdictions found either no change or increased cannabis use whereas a higher proportion of those studies conducted in illegal jurisdictions found decreased cannabis use during the pandemic. This finding may be explained by the pandemic creating a greater disruption in illegal markets during the pandemic (e.g., due to stay-at-home orders) [32, 91–94] relative to legal markets, particularly in jurisdictions where cannabis stores were deemed essential services (e.g., in Canada, [46, 95]). However, there is an urgent need for more research focusing on the impact of public health measures as well as legalization policies on cannabis use.

Overall, the findings from this study can be used by health care and policy decision makers to understand the impact of the pandemic on cannabis use and the factors associated with change in use. Findings may aid policy makers in developing strategies to inform the general population about risks of cannabis related harms, to address and ultimately prevent the consequences of increased cannabis consumption, and reduce the burden on the healthcare systems during pandemics. A few strategies for public policy decision makers include continued monitoring of cannabis use during and post pandemic, public guidance about prevention or moderation of cannabis use and implementation of measures to address the impacts of increased cannabis use [96].

This scoping review has several limitations. Only studies in English that included participants 18 years or older were included in this review which limits the generalizability of findings. The lack of representativeness of the data in the studies also limits its generalizability. There were several important topics that were excluded from this scoping review including examinations of cannabis use as a predictor [97–99], a treatment option for COVID-19 [100–102], and proxy measures of cannabis use (e.g., waste water measurements, emergency department visits) [103–105]). These topics were outside the scope of this review, but would be valuable as separate reviews in their own right. The studies that were included in this review were based on self-reported cannabis use data which can be influenced by recall and social desirability biases. It is possible that some studies were missed or that some studies that were identified may not be publicly available. To reduce the number of studies excluded, we implemented a standardized process to reach authors to request articles. Additionally, the criteria used to categorize change in cannabis use could potentially impact the findings of this review. Although we categorized changes in cannabis use as increased, no change, or decreased, the same study may have reported all three changes and thus could be interpreted in different ways; however, utilizing an organized approach helped synthesize and report the findings. Since the established scoping review methodology by Arksey and O’Malley (2005) [19] was use in this study, we did not conduct a quality assessment of included studies. Stakeholder consultations were not conducted for this scoping review since the focus was on self-reported change in cannabis use during the pandemic.

## Conclusion

Cannabis use has been impacted by the COVID-19 pandemic due to several associated factors. The information from this review sheds light on potentially concerning patterns of cannabis use following global health emergencies and can be utilized by healthcare workers and decision makers to support people who use cannabis and to prevent cannabis related harms during future pandemics. Further research is needed to understand the change in specific types and modes of cannabis use, as well as the impact of public health policies on cannabis use.

**Table 1:** Documents reporting change in cannabis use based on population group

Population group (Number of documents)	Change in cannabis use	N (%)	References
General population (33)	Increase	15 (45.5)	[7, 9, 10, 12, 14, 15, 52, 57, 67, 74, 106–110]
	No change	15 (45.5)	[13, 16, 26, 54, 56, 58–61, 71, 75, 76, 79, 80, 111]
	Decrease	3 (9.0)	[8, 11, 112]
Youth/young adults/emerging adults/students (41)	Increase	18 (43.9)	[5, 36, 37, 53, 64, 83, 113–124]
	No change	14 (34.1)	[39, 40, 42, 49, 62, 63, 68, 93, 95, 125–129]
	Decrease	9 (22.0)	[34, 38, 44, 65, 69, 70, 130–132]
People who use cannabis (14)	Increase	7 (50.0)	[17, 27, 28, 31–33, 82]
	No change	6 (42.9)	[4, 29, 30, 41, 72, 133]
	Decrease	1 (7.1)	[91]
People with health/mental health comorbidities (10)	Increase	5 (50.0)	[35, 46, 55, 73, 134]
	No change	5 (50.0)	[135–139]
	Decrease	0 (0)	
People who use substances (17)	Increase	10 (58.8)	[43, 50, 77, 94, 140–145]
	No change	2 (11.8)	[47, 146]
	Decrease	5 (29.4)	[45, 84, 89, 147, 148]
Occupational (7)	Increase	4 (57.1)	[51, 92, 149, 150]
	No change	2 (28.6)	[151, 152]
	Decrease	1 (14.3)	[153]
Sexual minority (7)	Increase	4 (57.1)	[6, 48, 78, 81]
	No change	2 (28.6)	[66, 154]
	Decrease	1 (14.3)	[155]

### Electronic supplementary material

Below is the link to the electronic supplementary material.

Supplementary Material 1

Supplementary Material 2

Supplementary Material 3

## Data Availability

The dataset created and used during the current study are available from the corresponding author on reasonable request.
