# Supplementary material for: Changes in self-reported cannabis use during the COVID-19 pandemic: a scoping review
Source: BMC Public Health. 2023 Nov 1;23:2139. doi: 10.1186/s12889-023-17068-7 (PMC10621278; doi:10.1186/s12889-023-17068-7)
Supplement: Supplementary file 2 — Supplementary Material 2 [file 12889_2023_17068_MOESM2_ESM.pdf]

Supplementary Material 2

SEARCH STRATEGIES

Medline Search

1. exp "Marijuana Use"/
2. exp Marijuana Abuse/
3. exp Medical Marijuana/
4. exp Cannabinoids/
5. exp Cannabaceae/
6. (marijuana$ or marihuana$).mp.
7. (Hashish$ or hash$ or bhang$ or ganja$ or ganjah$ or hemp$ or charas$).mp.
8. Cannabi$.mp.
9. canabi$.mp.
10. (THC$ or CBD$ or AEA).mp.
11. (cannador$ or eucannabinolide$ or 8001-45-4 or 8063-14-7 or 38458-58-1 or delta3-thc$ or delta3thc$ or delta-9-THC$ or delta9THC$ or sp-104 or sp104 or 5957-75-5 or 1972-08-3 or Deltanyne$ or Dronabinol$ or Marinol$ or dronabinolum$ or ea-1477 or ea1477 or tetranabinex$ or qcd-84924 or qcd84924 or 7663-50-5 or 9tetrahydrocannabinol$ or nabidiolex$ or 13956-29-1 or delta9$11$tetrahydrocannabinol$ or Tetrahydrocannabinol$ or tetra-hydrocannabinol$ or endocannabinoid$ or dexanabinol$ or Hu-210 or Hu-211 or hu210 or hu211 or 112924-45-5 or Nabilone$ or Cesamet$ or cesametic$ or cpd109514 or cpd-109514 or lilly-109514 or lilly109514 or 51022-71-0 or Nabiximols$ or Sativex$ or Gw-1000 or gw1000 or sab-378 or sab378 or 56575-23-6 or nantradol$ or cp-44001 or cp44001 or cp-44001-1 or cp 440011 or cp440011 or cp44001-1 or 72028-54-7 or ea-1477 or ea1477 or tetranabinex$ or qcd-84924 or qcd84924 or 7663-50-5).mp.
12. exp COVID-19/
13. covid$.mp.
14. nCov$.mp.
15. ncovid$.mp.
16. n covid$.mp.
17. CoV2$.mp.
18. CoV 2$.mp.
19. COVID19$.mp.

20. COVID2019$.mp.

21. COVID 19$.mp.

22. COVID 2019$.mp.

23. (Novel adj3 Coronavirus$).mp.

24. (Novel adj3 Corona virus$).mp.

25. (novel adj3 coronaravirus$).mp.

26. (novel adj3 coronara virus$).mp.

27. SARS COV 2$.mp.

28. SARS COV2$.mp.

29. Severe Acute Respiratory Syndrome Corona$ 2$.mp.

30. (coronavirus$ adj3 disease$ adj3 2019$).mp.

31. (coronavirus$ adj3 disease$ adj3 19$).mp.

32. (corona virus$ adj3 disease$ 2019).mp.

33. (corona virus$ adj3 disease$ 19$).mp.

34. SARS Corona$ 2.mp.

35. SARS CoV2$.mp.

36. Severe Acute Respiratory Syndrome Corona virus$ 2$.mp.

37. Severe Acute Respiratory Syndrome CoV 2$.mp.

38. Severe Acute Respiratory Syndrome CoV2$.mp.

39. HCoV 19$.mp.

40. novel cov$.mp.

41. or/1-10 [cannabis set]

42. or/12-38 [covid-19 set]

43. 41 and 42

44. limit 43 to yr="2020 -Current"

Grey Literature Search

Preprint Servers

bioRxix

https://www.biorxiv.org/

medRxix

https://www.medrxiv.org/

OSF Preprints

https://osf.io/preprints/

List of Governmental Agencies/ Other Organizations Searched

International

United Nations Office on Drugs and

Crime (UNODC)

COVID-19 and the drug supply chain:

from production and trafficking to use

https://www.unodc.org/documents/data-and-analysis/covid/Covid-19-and-drug-supply-chain-Mai2020.pdf

Europe

European Monitoring Centre for Drugs

and Drug Addiction (EMCDDA)

European Web Survey on Drugs

https://www.emcdda.europa.eu/news/2022/1/latest-european-web-survey-drugs-finds-cannabis-and-ecstasy-use-most-impacted-covid-19_en

Australia

National Drug Strategy Household Survey

https://campaigns.health.gov.au/drughelp/drug-trends-and-statistics

Impact of the COVID-19 pandemic on cannabis demand and supply in Australia

https://www.aic.gov.au/publications/sb/sb33

National Drug & Alcohol Research Centre Medicine ADAPT Study

https://ndarc.med.unsw.edu.au/resource/key-findings-australians-drug-use-adapting-pandemic-threats-adapt-study

https://ndarc.med.unsw.edu.au/resource/key-findings-australians-drug-use-adapting-pandemic-threats-adapt-study-wave-2

UNSW Social Policy Research Centre

van de Ven, K., Ritter, A., & Stirling, R. (2021). The impact of the COVID-19 pandemic on the non-government alcohol and other drug sector. DPMP Monograph No. 34. Sydney: UNSW Social Policy Research Centre

http://unsworks.unsw.edu.au/fapi/datastream/unsworks:74035/binaba0ec82-1837-40d4-9570-4ab50ee3217c?view=true&xy=01

Alcohol and Drug Council of Australia

(ADCA)

https://aadc.org.au

Australian National Council on Drugs

(ANCD)

https://idpc.net/publications

Drug Policy Modelling Program (DPMP)

https://ndarc.med.unsw.edu.au/resources

Australian Drug Trends 2020: Key Findings from the National Illicit Drug Reporting System (IDRS) Interviews

https://ndarc.med.unsw.edu.au/resource/australian-drug-trends-2020-key-findings-national-illicit-drug-reporting-system-idrs

Australian Drug Trends 2020: Key Findings from the National Ecstasy and Related Drugs Reporting System (EDRS) Interviews

https://ndarc.med.unsw.edu.au/resource/australian-drug-trends-2020-key-findings-national-ecstasy-and-related-drugs-reporting

Canada

Statistics Canada: Cannabis Statistics Hub at https://www150.statcan.gc.ca/n1/pub/13-610-x/13-610-x2018001-eng.htm

Statistics Canada

Alcohol and cannabis use during the pandemic: Canadian Perspectives Survey Series

https://www150.statcan.gc.ca/n1/daily-quotidien/210304/dq210304a-eng.htm

Health Canada/Statistics Canada

Canadian Alcohol and Drugs Survey

https://www.canada.ca/en/health-canada/services/canadian-alcohol-drugs-survey.html

CCSA

COVID-19, Alcohol and Cannabis Use

https://www.ccsa.ca/covid-19-alcohol-and-cannabis-use-report

The Mental Health Commission of Canada

Mental Health and Substance Use During COVID-19

https://www.mentalhealthcommission.ca/wp-content/uploads/drupal/2021-02/mhcc_ccsa_covid_leger_poll_eng.pdf

Ireland

Government of Ireland

Impact of COVID-19 on Drug and Alcohol

Services and People who use Drugs in

Ireland: A report of survey findings

https://igees.gov.ie/wp-content/uploads/2021/01/Covid-Rapid-Impact-Assessment.pdf

DrugNet Ireland

Impact of Covid-19 on drug use in Ireland

https://www.hrb.ie/fileadmin/2._Plugin_related_files/Publications/2021_publications/2021_HIE/Evidence_Centre/Drugnet_Ireland_76.pdf

National Drug and Alcohol Survey (NDAS) 2019-2020

https://www.hrb.ie/publications/publication/the-2019-20-irish-national-drug-and-alcohol-survey-main-findings/returnPage/1/

New Zealand

Government of New Zealand, Ministry of Health

Drug Use and Statistics

https://www.health.govt.nz/nz-health-statistics/health-statistics-and-data-sets/drug-use-data-and-stats

Drug Foundation

Pulse survey

https://www.drugfoundation.org.nz/assets/uploads/2020-uploads/Covid-19-resources/Pulse-survey-of-addiction-services-and-people-who-use-drugs-during-alert-level-4.pdf

Impact of Covid-19 lockdown on youth

Alcohol and other drug (AOD) use : Findings from a survey of AOD services in New Zealand

https://www.drugfoundation.org.nz/assets/uploads/2020-uploads/Covid-19-impact-on-youth-AOD-use-summary.pdf

United Kingdom

Government of UK

Collection of Drug Misuse Statistics

https://www.gov.uk/government/collections/drug-misuse-declared

Aldridge, J., Garius, L., Spicer, J., Harris, M., Moore, K. & Eastwood, N. (2021) Drugs in the Time of COVID: The UK Drug Market Response to Lockdown

Restrictions, London: Release

https://www.release.org.uk/sites/default/files/pdf/publications/Release%20COVID%20Survey%20Interim%20Findings%20final.pdf

Department of Health, Northern Ireland

Statistics from the Northern Ireland Substance Misuse Database: 2019/20

https://www.health-ni.gov.uk/news/statistics-northern-ireland-substance-misuse-database-201920

Northern Ireland, Statistics and Research Agency

https://www.nisra.gov.uk/statistics

Scottish Schools Adolescent Lifestyle and Substance Use Survey (SALSUS): drug use report 2018

https://www.gov.scot/publications/scottish-schools-adolescent-lifestyle-substance-use-survey-salsus-drug-use-report-2018/

Government of Scotland

COVID-19 impact on drug related abuse in Scotland

https://www.gov.scot/publications/foi-202100174213/

ScotPHO

Drug Misuse

https://www.scotpho.org.uk/behaviour/drugs/data/availability-and-prevalence

USA

CDC

Behavioural Risk Factor Surveillance System (BRFSS)

https://www.cdc.gov/chronicdisease/resources/publications/factsheets/brfss.htm

Youth Risk Behavior Surveillance System (YRBSS)

https://www.cdc.gov/healthyyouth/data/yrbs/index.htm

National Health and Nutrition Examination Survey

https://www.cdc.gov/nchs/nhanes/index.htm

NIDA

Community Epidemiology Work Group

(CEWG) (part of the National Institute

on Drug Abuse - NIDA)

https://nida.nih.gov/about-nida/organization/divisions/division-epidemiology-services-prevention-research-despr/community-epidemiology-work-group-cewg

Monitoring the Future

https://nida.nih.gov/drug-topics/trends-statistics/monitoring-future

US Department of Transportation, NHTSA

Drug and Alcohol Prevalence in Seriously and Fatally Injured Road Users Before and During the COVID-19 Public Health Emergency

From https://rosap.ntl.bts.gov/view/dot/50941

National Centre for Health Statistics

(NCHS)

https://www.cdc.gov/nchs/index.htm

SAMHSA

Key Substance Use and Mental Health Indicators in the United States: Results from the 2020 National Survey on Drug Use and Health

https://www.samhsa.gov/data/sites/default/files/reports/rpt35325/NSDUHFFRPDFWHTMLFiles2020/2020NSDUHFFR1PDFW102121.pdf
