# Supplementary material for: Changes in self-reported cannabis use during the COVID-19 pandemic: a scoping review
Source: BMC Public Health. 2023 Nov 1;23:2139. doi: 10.1186/s12889-023-17068-7 (PMC10621278; doi:10.1186/s12889-023-17068-7)
Supplement: Supplementary file 3 — Supplementary Material 3 [file 12889_2023_17068_MOESM3_ESM.docx]

**Supplementary Material 3**

**Table A: Characteristics of documents included in the scoping review**

| **Author (year)**  **Country** | **Study design**  **Methods** | **Sample size**  **Age: M (SD)/Median/Modal, Range, Groups N (%)**  **Sex or gender: N (%)** | **Race/Ethnicity: N (%)** | **Target population** | **Legal status of cannabis** | **Level of public health measures** | **Type of cannabis use** | **Mode of consumption** |
| --- | --- | --- | --- | --- | --- | --- | --- | --- |
| Armour *et al.* (2022) [1]  International study | Cross-sectional  Online survey | N=1634  30.7 (7.1)  Not specified | Not specified | People with health/mental health comorbidities | Variable | Not specified | Medical,  Recreational non-medical | Oils  Oral capsules  Smoking  Suppositories  Vaping |
| Assaf *et al.* (2022) [2]  USA | Cross-sectional  Online survey | N=1886  33.7 (8.8)  Females: 666 (35.3%) Males: 1189 (63.0%)  Other: 2 (0.1%) | Hispanic/Latino: 511 (27.1%)  Non-Hispanic Asian: 36 (1.9%)  Non-Hispanic Black: 202 (10.7%)  Non-Hispanic American Indian or Alaska Native: 51 (2.7%)  Non-Hispanic Native Hawaiian or Pacific Islander: 17 (0.9%)  Non-Hispanic White: 999 (53.0%)  Non-Hispanic Other: 24 (1.3%)  Unknown: 46 (2.4%) | People who use cannabis | Variable | Cannabis dispensaries deemed essential businesses | Medical,  Recreational non-medical | Dabbing  Edibles  Oil  Smoking  Topicals  Vaping |
| Bartel *et al.* (2020) [3]  Canada | Longitudinal  Online survey | N=70  22.0  Females: 45 Males: 24 Other: 1 | Not specified | Youth/young adults/students | Legal | State of emergency  Stay-at-home orders | Recreational non-medical | Edibles  Smoking  Vaping |
| Baum *et al.* (2022) [4]  USA | Cross-cohort  Online survey | N=1430  Median=46  Males: 1100 (77.0%) | Black, non-Hispanic: 826 (57.8%) White, non-Hispanic: 160 (11.2%)  Hispanic: 363 (25.4%)  Other: 81 (5.6%) | People with health/mental health comorbidities | Not specified | Social isolation  Quarantine | Not specified | Not specified |
| Ben Salah *et al.* (2022) [5]  International study | Cross-sectional  Online survey | N=2907  38.7 (14.3)  Male: 870 (30%) Female: 2023 (69.8%) Other: 6 (0.2%) | Not specified | General population | Not specified | Self-isolation  Nationwide lockdowns  Restrictions on movement | Not specified | Not specified |
| Benschop *et al.* (2021) [6]  The Netherlands | Cross-sectional  Online survey | N=6070  29.1 (16.8)  Females: 49.5%  Males: 50.0%  Other: 0.5% | Not specified | General population | Not specified | Closure of public venues  Cancellation of major events  Suspension of contact (i.e., face to face) professions  Restriction on gatherings  Social distancing  Work-from-home orders | Not specified | Smoking |
| Blithikioti *et al.* (2021) [7]  Spain | Cross-sectional  Online survey | N=303  49.3 (15.6)  Females: 113 (37.3%) Males: 186 (61.4%) Non-binary/other: 4 (1.3%) | Not specified | Substance users | Not specified | Not specified | Not specified | Not specified |
| Bochicchio *et al.* (2021) [8]  USA | Descriptive Phenomenological Study  Online or phone interviews | N=16  53.5  Cisgender: 15 Non-binary: 1 | African American/Black: 8 Latinx: 5 White: 3 | Sexual minority | Legal | Stay-at-home orders | Not specified | Edibles  Smoking |
| Boehnke *et al.* (2021) [9]  USA | Cross-sectional  Online survey | N=353  37 (11) Females: 55.5%  Males: 43.9%  Other: 0.6% | White: 80.2%  Asian: 7.6%  Hispanic/Latino: 7.4%  Black/African American: 7.1%  American Indian or Alaska Native: 3.7%  Native Hawaiian/Pacific Islander: 0.6%  Other: 0.6% | People who use cannabis | Variable | Social distancing  Quarantine  Stay-at-home orders | Medical,  Recreational non-medical | Not specified |
| Bonar *et al.* (2021) [10]  USA | Pilot Randomized Controlled Trial  Online survey | N=141  21.1 (2.2)  Females: 80 (56.7%)  Males: 61 (43.3%) | White: 99 (70.2%)  Black/African American: 28 (19.9%)  Other: 14 (9.9%) | Youth/young adults/students | Variable | Stay-at-home orders  School closures | Not specified | Smoking  Vaping  Eating Dabbing Drinking Topicals |
| Bonny-Noach *et al.* (2021) [11]  Israel | Cross-sectional  Online survey | N=750  29.8 (11.8)  Females: 72.8% Males: 27.2% | Not specified | General population | Decriminalized | Not specified | Not specified | Not specified |
| Brenneke *et al.* (2022) [12]  USA | Longitudinal  Online survey | N=1761  18–29 years = 23.1%  30–49 years = 45.8%  50–64 years = 20.5%  65 + years = 10.6%  Females: 900 (51.1%)  Males: 861 (48.9%) | White: 990 (56.2%)  Black: 280 (15.9%)  Hispanic/Latinx: 340 (19.3%)  Other: 151 (8.6%) | General population | Variable | Stay-at-home orders  Closure of non-essential businesses  Remote work  Cannabis dispensaries declared essential businesses | Medical,  Recreational non-medical | Not specified |
| Brotto *et al.* (2021) [13]  Canada | Cross-sectional  Online survey | N=6076  Age (females): 48.5 (12.0)  Females: 5254 (86.4%)  Males: 750 (12.3%) Gender diverse: 72 (1.2%) | Females:  White: 4265 (81.2%)  Black: 28 (0.5%)  Chinese/Taiwanese: 311 (5.9%)  South Asian: 123 (2.3%)  Other ethnicity: 504 (9.6%) | General population | Not specified | Not specified | Not specified | Not specified |
| Busse *et al.* (2021) [14]  Germany | Cross-sectional  Online survey | N=5021  24.4 (5.1)  Females: 69.4% Males: 29.4% Diverse: 1.2% | Not specified | Youth/young adults/students | Not specified | Closure of universities, non-essential shops, and restaurants  Social distancing | Not specified | Not specified |
| Camacho-Rivera *et al.* (2021) [15]  International study | Cross-sectional  Online survey | N=158  58.1  Females: 68 (43.3%)  Males: 87 (55.4%) Other: 1 (0.6%) Transgender: 1 (0.6%) | Non-Hispanic White: 132 (85.2%) Non-Hispanic Black or African American: 6 (3.9%) Hispanic: 9 (5.8%)  Non-Hispanic Asian: 2 (1.3%)  Native Hawaiian or Other Pacific Islander: 1 (0.6%)  American Indian or Alaska Native: 5 (3.2%) | People with health/mental health comorbidities | Medical cannabis legal | Not specified | Medical,  Recreational non-medical | Smoking  Vaping |
| Carlyle *et al.* (2021) [16]  Australia | Cross-sectional  Online survey | N=325  36.7 (11.0)  Females: 185 (57%) | Indigenous Australians: 56 (17%) | People who use substances | Not specified | Not specified | Not specified | Not specified |
| Case *et al.* (2022) [17]  USA | Cross-sectional qualitative study  Online interviews | N=50  21.4 (1.9)  Males: 30 Female: 20 | Asian: 40% White non-Hispanic: 18%  Hispanic: 20% African American: 18% Another race: 4% | Youth/young adults/students | Illegal | Not specified | Not specified | Vaping |
| Chaiton *et al.* (2021) [18]  Canada | Cross-sectional  Online survey | N=6721  16 - 25 years  Males: 21% | White: 74% | Youth/young adults/students | Not specified | Not specified | Not specified | Not specified |
| Clendennen *et al.* (2021) [19]  USA | Cross-sectional  Online survey | N= 709  19.9 (1.5) Males: 234 (41.1%)  Females: 336 (59.0%) | Hispanic/Latino 227: (39.8%)  Non-Hispanic white: 177 (31.0%)  Non-Hispanic black: 86 (15.1%)  Non-Hispanic other: 81 (14.2%) | Youth/young adults/students | Not specified | Not specified | Not specified | Smoking |
| Conroy *et al.* (2021) [20]  USA | Cross-sectional  Online survey | N=834  43 (12.9)  Females: 656 (78.7%) Males: 173 (20.7%) Nonbinary: 2 (0.2%) Prefer not to say: 3 (0.4%) | Non-Latino: 798 (95.5%) Latino: 29 (3.5%) White: 712 (85.2%) Asian: 38 (4.5%) Black/African American: 24 (2.9%) More than one race: 30 (3.6%) | Occupational | Not specified | Stay-at-home orders  Transition to telemedicine for ambulatory healthcare services | Medical,  Recreational non-medical | Not specified |
| Cousijn *et al.* (2021) [21]  The Netherlands | Case-control  Online survey | N=183  Age range = 18 – 46 years Not specified | Not specified | People who use cannabis | Not specified | Social isolation  Stay-at-home orders  Work and school from home | Not specified | Not specified |
| Currie (2021) [22]  Canada | Cross-sectional  Online survey | N=933  18–34 years = 24.5%  35–54 years = 42.7%  55+ years = 32.8%  Females: 471 (50.5%) Males: 462 (49.5%) | Not specified | People with health/mental health comorbidities | Not specified | Cannabis dispensaries deemed essential  Work from home  Online school  Border closures | Not specified | Not specified |
| Das *et al.* (2022) [23]  USA | Longitudinal  Online survey | N=7597  18–34 years = 18.1% 35–44 years = 19.5% 45–54 years = 18.3% 55–64 years = 20.7% 65+ years = 23.4%  Females: 58.5% Males: 41.6% | White: 66.8% Black: 7.6% Hispanic: 15.2% Other: 10.5% | General population | Not specified | Stay-at-home orders  Closure of non-essential businesses  Travel restrictions | Not specified | Not specified |
| de Quervain *et al.* (2020) [24]  Switzerland | Repeated cross-sectional  Online survey | N=11167  41.9 (13.1)  Females: 72.1% Males: 27.1% | Not specified | General population | Not specified | Not specified | Not specified | Not specified |
| Dietz *et al.* (2022) [25]  Germany | Cross-sectional  Online survey | Baseline  N=4351  23.8 (4.4)  Females: 3065 (70.4%)  Males: 1246 (28.6%)  Diverse: 39 (0.9%) | Not specified | Youth/young adults/students | Illegal | Closure of universities  Online school | Not specified | Not specified |
| Donovan and Portman (2021) [26]  USA | Cross-sectional  Online survey | N=85  Using cannabis:  54.1 (14.9)  Females: 10 (38.5%)  Males: 16 (61.5%) | Using cannabis:  White race: 24 (92.3%)  Non-white race: 2 (7.7%)  Non-Hispanic: 26 (100.0%)  Hispanic: 0 (0.0%) | People with health/mental health comorbidities | Not specified | Not specified | Medical | Not specified |
| Dozois (2021) [27]  Canada | Cross-sectional  Online survey | N=1803  47.9 (17.2)  Females: 927 Males: 867 Another gender: 7 | Not specified | General population | Not specified | Not specified | Not specified | Not specified |
| Dumas *et al.* (2022) [28]  Canada | Longitudinal  Online survey | N=1068  Baseline:  17.0 (0.8)  Females: 76.7%  Males: 21%  Other: 2.3% | White/European: 65.2% Asian: 16.3% Black North American/African: 3.9% Latino: 3.1% Other: 11.6% | Youth/young adults/students | Not specified | Stay-at-home orders  Closure of non-essential businesses  Ban on social gatherings | Not specified | Not specified |
| Dyar *et al.* (2021) [29]  USA | Longitudinal  Online survey | N=212  22.4 (2.1)  Cisgender Women: 156 (73.6%) Genderqueer/Non-Binary: 38 (17.9%) Another Identity: 18 (8.5%) | White: 86 (40.6%) Black: 37 (17.5%) Latinx: 52 (24.5%) Other Race/Ethnicity: 37 (17.5%) | Sexual minority | Not specified | Not specified | Not specified | Not specified |
| El-Gabalawy and Sommer (2021) [30]  Canada | Cross-sectional  Online survey | Baseline:  N=4627  15 -34 years = 31.0% 35-54 years = 31.3% 55-74 years = 31.6% 75+ years = 6.1%  Females: 50-57% Males: 44-50% | Not specified | General population | Not specified | Travel restrictions  Quarantine  Closure of non-essential businesses  Physical distancing | Not specified | Not specified |
| Fedorova *et al.* (2021) [31]  USA | Longitudinal mixed methods study  Online surveys and interviews | Quantitative sample: N=158  Qualitative sample: N=29  27.2 (2.4)  Males: 102 (64.6%) | Hispanic/Latinx: 78 (49.4%)  Non-Hispanic race White: 40 (25.3%)  Black/African American: 26 (16.5%) Multi-Racial: 10 (6.3%) Asian/Pacific Islander: 1 (0.6%) | People who use cannabis | Not specified | Stay-at-home orders | Medical,  Recreational non-medical | Not specified |
| Fernández-Artamendi *et al.* (2021) [32]  Spain | Cross-sectional  Online survey | N=89  29.0 (9.3)  Males: 73% | Not specified | People who use cannabis | Illegal | Quarantine  Closure of non-essential businesses  Stay-at-home orders | Not specified | Marijuana Hashish Vaping  Spice |
| Firkey *et al.* (2020) [33].  USA | Cross-sectional  Online survey | N=212  22.1 (2.1)  Females: 107 (50.5%)  Males: 105 | Caucasian/White: 150 (70.8%)  Black/African American: 16 (17.5%) Asian/Pacific Islander: 31 (14.6%) Mixed race/other: 11 (5.2%)  Hispanic or Latino: 36 (17.0%)  Non-Hispanic or Latino: 175 (82.5%) | Youth/young adults/students | Not specified | Closure of non-essential businesses  Closure of universities  Restrictions on movement, gatherings, social activities | Not specified | Not specified |
| Fitzke *et al.* (2021) [34]  USA | Repeated cross-sectional  Online survey | N=1025  34.6 (3.5)  Females: 96 (9.4%) Males: 917 (89.5%) Other: 12 (1.2%) | Hispanic/Latinx: 90 (8.8%) Black/African American: 62 (6.0%) Asian: 12 (1.2%) White: 850 (82.9%) Multiracial/other: 11 (1.1%) | Occupational | Not specified | Not specified | Not specified | Edibles  Smoking  Vaping |
| Gaiha *et al.* (2020) [35]  USA | Cross-sectional  Online survey | N= 2167  19.17 (2.3)  Females: 1397 (64.5%)  Males: 723 (33.4%) Other/nonbinary: 46 (2.1%) | African American/Black: 319 (14.7%)  Asian/Pacific Islander, non-Hispanic: 146 (6.7%)  Hispanic: 416 (19.2%)  White, non-Hispanic: 1102 (50.9%)  Other/multiracial: 184 (8.5%) | Youth/young adults/students | Not specified | Stay-at-home orders  Social distancing | Not specified | Edibles  Smoking  Vaping |
| Gattamorta *et al.* (2021) [36]  USA | Cross-sectional  Online survey | N=2319  44.3 (15.5)  Females: 1045 (45.2%) Males: 1243 (53.7%) Other: 11 (0.5%) Transgender: 14 (0.6%) | Non-Hispanic White: 1740 (75%) Non-Hispanic Black: 99 (4.3%) Hispanic: 301 (12.9%) Other: 163 (7%) | People who use cannabis | Not specified | Not specified | Medical | Not specified |
| Gaume *et al.* (2021) [37]  Switzerland | Cross-sectional Mixed methods, exploratory  Paper and pencil surveys (posted by mail) and phone interviews | Quantitative: N = 100  Qualitative: N=31  Range = 18–59 years  Females: 25% | Not specified | People who use substances | Illegal | Closure of non-essential businesses  Closure of schools  Ban on large gatherings | Not specified | Herbal cannabis (dried flower) Resin (hashish) |
| Glazer and Vallis (2022) [38]  International study | Cross-sectional  Online survey | Survey:  N=1098  58 (10.6)  Females: 550 (50.8%) | Not specified | People with health/mental health comorbidities | Not specified | Not specified | Not specified | Not specified |
| Goodyear *et al.* (2021) [39]  Canada | Repeated cross-sectional  Online survey | N=502  18–34 years = 28.3% 35–54 years = 41.8% 55+ years = 29.9%  Cisgender woman: 38.6% Cisgender man: 54.8% Trans man: 0.8% Trans woman: 1.0% Non-binary: 3.2% Two-Spirit 2: 1.0% Not listed: 0.4% | Non-racialized: 334 (69.2%) Racialized (non-Indigenous): 123 (25.5%) Indigenous: 26 (5.4%) | Sexual minority | Not specified | Not specified | Not specified | Not specified |
| Graupensperger *et al.* (2021) [40]  USA | Longitudinal  Online survey | N=572  25.14 (1.84)  Females: 60.8% | White/Caucasian: 50.6% Asian/Asian American: 17.5% Other/Non-Hispanic: 23.8% Hispanic: 8.2% | Youth/young adults/students | Variable | Not specified | Not specified | Not specified |
| Gritsenko *et al.* (2021) [41]  International study | Cross-sectional  Online survey | N=939  21.8 (5.4)  Males: 180 (19.2%) Females: 757 (80.8%) | Not specified | Youth/young adults/students | Not specified | Quarantine  Restrictions on movement Social distancing  Use of masks and gloves  Ban on public events  Closure of border and air traffic | Not specified | Not specified |
| Hicks *et al.* (2022) [42]  USA | Longitudinal  Online survey | N=323  Not specified  Not specified | Individuals self-identified as Black, Asian, Hispanic/Latinx, and more than one race | Youth/young adults/students | Illegal | Not specified | Not specified | Not specified |
| Hochstatter *et al.* (2021) [43]  USA | Cohort  Online survey | N=64  Not specified  Males: 48 (75%) | White: 22 (34%) Black or African American: 38 (59%) Mixed: 1 (2%) Other: 3 (5%) Hispanic or Latino: 6 (9%) | People with health/mental health comorbidities | Not specified | Closure of schools  Ban on large gatherings  Stay-at-home orders  Closure of non-essential businesses | Not specified | Not specified |
| Howard *et al.* (2021) [44]  USA | Cross-sectional  Online survey | N=2267  47.8 (12.9)  Females: 87.8% | White: 89.9% Black: 0.9% Hispanic: 4.9% Other/unspecified: 4.3% | General population | Not specified | Social distancing  Stay at home orders  Work and school from home | Not specified | Not specified |
| Imboden *et al.* (2021) [45]  Switzerland | Cross-sectional  Online survey | N=275  24.8 (6.0)  Females: 97 (33.7%) Males: 178 (61.8%) | Not specified | Occupational | Illegal | Travel bans  Ban on large gatherings  Stay at home orders  Closure of all sports facilities | Not specified | Not specified |
| Imtiaz *et al.* (2021) [46]  Canada | Repeated cross-sectional  Online survey | N=369 18–29 years: 21.7%  30–49 years: 49.3%  ≥50 years: 29.0%  Females/Other: 46.9% Males: 53.1% | Non-White: 113 (31.1%)  White: 250 (68.7%) | General population | Legal | Physical distancing  Closures of schools  Work from home  Cancellation of public gatherings and events | Not specified | Not specified |
| Imtiaz *et al.* (2022) [47]  Canada | Cross-sectional  Online survey | Baseline  N = 1005  18 to 29 years = 13.1%  30 to 39 years = 26.1%  40 to 49 years = 12.8% 50 to 59 years = 17.6%  60 to 69 years = 19.1%  70+ years = 11.2%  Females: 498 (49.7%)  Males: 504 (50.3%) | Not specified | General population | Legal | Closures of non-essential business  Restrictions on gatherings  Work from home orders | Not specified | Not specified |
| Janulis *et al.* (2021) [48]  USA | Longitudinal  In person surveys | N=458  21.3  Males: 93.2%  Transgender female: 3.3 % Another gender identity: 3.5 % | Not specified | Sexual minority | Legal | Not specified | Not specified | Not specified |
| Jodczyk *et al.* (2022) [49]  Poland | Cross-sectional  Online survey | N=1323  22.2 (4.2)  Females: 1021 (77.2%) Males: 297 (22.5%) Did not specify gender: 5 (0.4%) | Not specified | Youth/young adults/students | Illegal | Stay-at-home orders  Quarantine  Closures of basic facilities and services | Not specified | Not specified |
| Knell *et al.* (2020) [50]  USA | Cross-sectional  Online survey | N=1809  18–34 years = 31.5% 35–49 years = 39.8% 50+ years = 28.7%  Females: 1220 (67.4%) Males: 589 (32.6%) | Non-Hispanic white: 1483 (82.0%) Non-white: 326 (18.0%) | General population | Not specified | Stay-at-home orders  Ban on large gatherings  Restricted access to parks and community resources  Closure of schools and non-essential businesses  Quarantine | Not specified | Not specified |
| Lake *et al.* (2022) [51]  USA | Cross-sectional  Online survey | N=598  Not specified  Not specified | Not specified | People who use cannabis | Variable | Stay-at-home orders | Medical,  Recreational non-medical | Dabbing  Edibles  Smoking  Topicals  Vaping |
| Lewis and Sznitman (2022) [52]  Israel | Cross-sectional  Online survey | N=440  26.8 (6.2)  Females: 45 (10.3%) Males: 394 (89.7%) | Not specified | People who use cannabis | Not specified | Not specified | Recreational non-medical | Not specified |
| Lin (2022) [53]  Canada | Cross-sectional  Online survey | N=3769  15–25 years = 159  25–44 years = 1145  45–64 years = 1472  ≥65 years = 993  Females: 2016 (50.7%) Males: 1753 (49.3%) | Not specified | General population | Not specified | Not specified | Not specified | Not specified |
| Lintzeris *et al.* (2021) [54]  Australia | Pre-post cohort  Electronic medical records, phone interviews and in person interviews | N=429  43 (10)  Females: 33% | Indigenous: 18% | People who use substances | Illegal | Ban on large gatherings Closure of non-essential businesses  Stay-at-home orders | Not specified | Not specified |
| Lukács (2021) [55]  Hungary | Cross-sectional  Online survey | N=421  26.6 (8.5)  Females: 67.5% Males: 31.6% Others: 1.0% | Not specified | Youth/young adults/students | Not specified | Self-isolation  Social distancing  Closure of universities  Online school | Not specified | Not specified |
| MacEachern *et al.* (2021) [56]  Canada | Cross-sectional  Online survey | N=12344  18–24 years = 10%  25–44 years = 35%  45–64 years = 33% 65+ years = 22%  Females: 7063 Males: 5255 | Member of racialized group Yes: 22-26% No: 74-78% | General population | Legal | Not specified | Not specified | Not specified |
| Manthey *et al.* (2021) [57]  International study | Cross-sectional  Online survey | N=36538  18–34 years = 33.5%  35–54 years = 43.4%  55+ years = 23.1%  Females: 51.6%  Males: 47.8%  Other: 0.6% | Not specified | People who use substances | Variable | Not specified | Not specified | Not specified |
| Meanley *et al.* (2022) [58]  USA | Longitudinal  Phone interviews | N=2121  53.5 (10.1)  Females: 1321 (62.3%) Males: 800 (37.7%) | Non-Hispanic White: 528 (26.4%)  Non-Hispanic Black: 1124 (56.1%)  Hispanic All Races: 297 (14.8%)  Non-Hispanic Other: 53 (2.7%) | People with health/mental health comorbidities | Not specified | Restrictions on social contact  Physical distancing | Therapeutic,  Recreational non-medical | Not specified |
| Merrill *et al.* (2022) [59]  USA | Longitudinal  Online survey | N = 223  21.3 (0.8)  Females: 61% | White: 63% Asian: 14%  Black: 5% Another race: 8% Bi- or multiracial 9% Hispanic/Latinx: 12% | Youth/young adults/students | Variable | Closure of colleges | Not specified | Concentrates  Edibles  Leaf |
| Mezaache *et al.* (2022) [60]  France | Cross-sectional  Online survey | N=4019  18 to 25 years = 44.4% 26 to 45 years = 45.5% >45 years = 10.0%  Females: 1045 (26.0%) | Not specified | People who use cannabis | Illegal | Closure of schools and non-essential businesses  Stay-at-home orders  Travel restrictions | Self-medication (categorized as Medical) | Edibles  Smoking  Vaping |
| Miech *et al.* (2021) [61]  USA | Longitudinal  In person and online surveys | Baseline: N=3770  Modal age = 18 (56%)  Females: 51% | Not specified | Youth/young adults/students | Not specified | Social distancing | Not specified | Not specified |
| Miller *et al.* (2022) [62]  USA | Longitudinal  Online survey | N=67  35.1 (11.3)  Females: 31 (46.3%) Males: 32 (47.8%) Non-binary/other gender: 3 (4.1%) Missing: 1 | Not specified | People who use cannabis | Legal | Stay-at-home orders | Not specified | Not specified |
| Mohr *et al.* (2021) [63]  USA | Cross-sectional  Online survey | N=215  24.8 (6.5)  Females: 68.1% Males: 22.9% Other: 9% | White: (66.1%) Non-white: (33.9%) | Youth/young adults/students | Legal | Closure of restaurants and bars  Ban on social events | Not specified | Not specified |
| Mora *et al.* (2020) [64]  USA | Cross-sectional  Phone surveys | N=1115  39.7 (12.6)  Female: 586 (52.6%) Male: 529 (47.4%) | Not specified | Occupational | Not specified | Not specified | Not specified | Not specified |
| Mravčík and Chomynová (2021) [65]  Czech Republic | Cross-sectional  Online survey | N=3000  Quota sampling by gender, age and region based on the structure of the Czech general adult population. | Not specified | General population | Not specified | Border closures  Closure of schools, shops and restaurants  Social distancing | Not specified | Not specified |
| Nguyen *et al.* (2021) [66]  USA | Cross-sectional  Online survey | N=4351  19.4 (2.6)  Females: 68.9% Males: 29.4% Other: 1.7% | Non-Hispanic White: 39.0% Non-Hispanic Black: 18.8% Hispanic: 26.2% Non-Hispanic API: 7.7% Non-Hispanic Other: 8.3% | Youth/young adults/students | Not specified | Not specified | Not specified | Edibles  Smoking  Vaping |
| Otiashvili *et al.* (2022) [67]  Georgia | Longitudinal, mixed methods study  Online survey and phone interviews | N=50  36 (9.9)  Females: 10 (20%) Male: 39 (78%) Nonbinary: 1 (2%) | Not specified | People who use substances | Illegal | Border closures  Restrictions on movement  Closure of businesses and educational institutions  Curfew | Not specified | Not specified |
| Palamar *et al.* (2020) [68]  USA | Cross-sectional  Online survey | N=128  23.3 (4.4)  Females: 79 (61.7%) Males: 49 (38.3%) | White: 53 (41.4 %) Black: 2 (1.6%) Hispanic: 7 (5.5%) Asian: 55 (43.0%) Other/Mixed: 11 (8.5%) | People who use substances | Not specified | Ban on nightlife  Stay-at-home order | Not specified | Not specified |
| Papp and Kouros (2021) [69]  USA | Repeated cross-sectional  Online survey | N=295  19.5 (0.7)  Females: 70.8% | Hispanic/Latinx: 7% White: 86.8%  American Indian/Alaska Native, Asian, Black or African American, Native Hawaiian or Pacific Islander, or reported multiple or other races: < 3%  Did not respond: 0.3% | Youth/young adults/students | Illegal | Limited access to residence halls  Online studies | Not specified | Not specified |
| Pavarin *et al.* (2022) [70]  Italy | Cross-sectional  Online interviews | N=183  28.7  Females: 33% | Not specified | People who use substances | CBD legal, Cannabis illegal | Social isolation  Stay-at-home orders  Closure of schools  Closure of public places  Closure of non-essential businesses | Not specified | CBD and smoking |
| Pedersen *et al.* (2021) [71]  USA | Longitudinal  Online survey | N=1025  34.6 (3.5)  Females: 96 (9.3%) Males: 917 (89.5%) Other: 12 (1.2%) | Hispanic/Latinx: 90 (8.8%) Black/African American: 62 (6.0%) Asian: 12 (1.2%) White: 850 (82.9%) Multiracial/other: 11 (1.1%) | Occupational | Variable | Cannabis dispensaries deemed essential businesses | Not specified | Edibles  Smoking Vaping |
| Pocuca *et al.* (2022) [72]  Canada | Longitudinal  Online survey | N=1096  21 - 22 years  Females: 62% | Not specified | Youth/young adults/students | Not specified | Social distancing  Work from home  Bans on indoor gatherings  Closure of schools and universities  Cannabis dispensaries deemed essential businesses | Not specified | Not specified |
| Potvin *et al.* (2022) [73]  Canada | Cross-sectional  Online survey | N=449  Young adults: 21.5 (2.1)  Females: 76.5% Males: 23.5% | Not specified | Youth/young adults/students | Not specified | Closure of public places, restaurants, and non-essential businesses  Ban on gatherings  Traveling restrictions  Closure of schools and universities | Not specified | Not specified |
| Rantis *et al.* (2021) [74]  Greece | Cross-sectional  Online survey | N=1043  43.0 (11.9)  Females: 807 (77.4%) Males: 223 (21.4%) Other: 3 (0.2%) Did not provide an answer: 10 | Not specified | General population | Not specified | Work from home  Reduced working hours or forced unemployment  Closure of non-essential businesses  Restrictions on social gatherings | Not specified | Not specified |
| Reilly *et al.* (2022) [75]  USA | Cross-sectional  Online survey | N=409  54.9 (16)  Males: 313 (76.5%)  Females: 94 (23%)  Transgender Male: 1 (0.2%)  Preferred not to answer: 1 (0.2%) | White: 370 (90.5%)  Black/African American: 22 (5.4%)  Other: 7 (1.7%) Asian: 5 (1.2%)  Native Hawaiian/Pacific Islander: 5 (1.2%)  American Indian/Alaska Native: 5 (1.2%)  Not Hispanic/Latino: 376 (91.9%)  Hispanic/Latino: 33 (8.1%) | Occupational | Not specified | Stay-at-home orders  Closure of non-essential businesses  Shifting clinical resources to COVID-19 specific care | Not specified | Not specified |
| Reuter *et al.* (2021) [76]  USA | Repeated cross-sectional and panel elements  Online survey | COVID survey:  N= 506  20.6 (4.8) Females: 83.8% Males: 16.0% No information: 0.2% | Not specified | Youth/young adults/students | Not specified | Stay-at-home orders  Reduced public transport  Mask mandates  Social distancing  Closure recreational facilities  Online or hybrid classes | Not specified | Not specified |
| Robillard *et al.* (2021) [77]  Canada | Case-control  Online survey | Clinical group:  N=1732  48.1 (15.6)  Females: 1284 (74.2%) | Clinical group: Caucasian: 1483 (85.6%) | General population | Not specified | Not specified | Not specified | Not specified |
| Rodriguez *et al.* (2022) [78]  International study | Cross-sectional  Online survey | N=160  57.8 Not specified | Non-Hispanic White: 129 (82.2%) Non-Hispanic Black or African American: 6 (3.8%)  Hispanic: 13 (8.3%) Asian: 3 (1.9%)  Other: 6 (3.8%) | People who use cannabis | Variable | Not specified | Medical | Not specified |
| Rogers *et al.* (2020) [79]  USA | Cross-sectional  Online survey | N=160  37.9 (11.2) Females: 43.5% | Not specified | General population | Variable | Not specified | Not specified | Not specified |
| Rolland *et al.* (2020) [80]  France | Cross-sectional  Online survey | N=11391  47.5 (17.3)  Females: 8782 (77.1%)  Males: 2557 (22.5%)  Other: 52 (0.5%) | Not specified | General population | Not specified | Closures of non-essential businesses  Stay-at-home orders | Not specified | Not specified |
| Romm *et al.* (2022) [81]  USA | Longitudinal  Online survey | N=1082  24.8 (4.7)  Females: 556 (51.4%)  Males: 494 (45.7%)  Other: 32 (3.0%) | White: 793 (73.3%) Black: 43 (4.0%)  Asian: 134 (12.4%)  Other: 112 (10.4%) Hispanic: 136 (12.6%) | Youth/young adults/students | Not specified | Not specified | Not specified | Not specified |
| Romm *et al.* (2022) [82]  USA | Longitudinal  Online survey | N=1082  24.8 (4.7)  Females: 556 (51.4%) | White: 793 (73.3%)  Black: 43 (4.0%)  Asian: 134 (12.4%)  Other: 112 (10.4%) | Youth/young adults/students | Not specified | Not specified | Not specified | Not specified |
| Salles *et al.* (2021) [83]  International study | Cross-sectional  Online survey | N=7084  42.3 (13.3)  Females: 4875 (69%)  Males: 2209 (31%) | Not specified | General population | Variable | Not specified | Not specified | Not specified |
| Salmon *et al.* (2022) [84]  Canada | Cross-sectional  Online survey | N= 664  Range = 16 - 21 years old  Not specified | Not specified | Youth/young adults/students | Not specified | Restrictions on public gatherings  Social/physical distancing  Closures of non-essential businesses  Closure of schools  Mask mandates | Not specified | Not specified |
| Sande *et al.* (2021) [85]  Slovenia | Cross-sectional  Online survey | N= 680  25  Females: 58.7% Males: 40.6% Other: 0.7% | Not specified | People who use substances | Illegal | Cessation of public transport  Closure of schools  Ban on public events  Closure of day centers  Reduction in psychosocial assistance | Not specified | Not specified |
| Schepis *et al.* (2021) [86]  USA | Cross-sectional  Online survey | N=4979  19.9 (3.5)  Females: 70.1 % | White: 48.5 % Black: 13.4 % Hispanic/Latinx: 6.3 % Asian: 6.9 % Native Hawaiian or Pacific Islander: 0.1 % American Indian or Alaska Native: 0.5 % Other: 0.9 % Multi-racial: 23.4 % | Youth/young adults/students | Not specified | Not specified | Not specified | Not specified |
| Scherbaum *et al.* (2021) [87]  Germany | Cross-sectional  Online survey | N=337  38.5 (10.3)  Males: 262 (77.8%) | Not specified | People who use substances | Illegal | Travel restrictions | Not specified | Not specified |
| Shapiro *et al.* (2022) [88]  Israel | Cross-sectional  Online survey | N=1020  16.7 (1.0)  Females: 57.3% Males: 42.7% | Not specified | Youth/young adults/students | Illegal | Closure of schools  Online studies  Restrictions on movement  National curfew | Not specified | Smoking |
| Sharma *et al.* (2020) [89]  USA | Cross-sectional  Online survey | N=1018  22.0 (2.1)  Females: 436 (80.4%) Males: 106 (19.6%) | Not specified | Youth/young adults/students | Not specified | Stay-at-home orders  Social distancing | Not specified | Not specified |
| Slemon *et al.* (2022) [90]  Canada | Repeated cross-sectional  Online survey | Baseline: N=2984 18-34 years = 9.7%  35-54 years = 42.4%  55+ years = 47.9%  Cisgender man: 1367 (45.4%)  Cisgender woman: 1598 (53.1%) Trans man: 4 (0.1%)  Trans woman: 17 (0.6%)  Non-binary: 9 (0.3%)  Two-Spirit: 6 (0.2%) | Non-racialized: 2142 (74.1%)  Racialized: 660 (22.8%)  Indigenous: 90 (3.1%) | Sexual minority | Not specified | Not specified | Not specified | Not specified |
| Somé *et al.* (2022) [91]  Canada | Repeated cross-sectional  Online survey | N=6021  18-39 years = 39.0%  40-59 years = 30.6% 60+ years = 30.4%  Males: 2986 (49.6%) Females: 2986 (49.6%) | Non-White: 1698 (28.2%) White: 4179 (69.4%) | General population | Not specified | Stay-at-home orders  Ban on large gatherings  Physical distancing  Self-isolation  Quarantine  Online school  Closure of non-essential businesses | Not specified | Not specified |
| Somé *et al.* (2022) [92]  Canada | Repeated cross-sectional  Online survey | N= 6016 18-39 years = 2334 40-59 years = 1835 60+ years = 1828 Transgender and gender-diverse individuals: 39 (0.7%) Cisgender individuals (men and women): 5964 (99.3%) | Asian: 1112 Black/ Indigenous/ Arab/Latino and other ethnicities: 567 White: 4170 | Sexual minority | Not specified | Not specified | Not specified | Not specified |
| Starks *et al.* (2020) [93]  USA | Cohort-control  Online survey | N=910  40.5 (13.4)  Not specified | Black: 261 (28.7)  Latino: 126 (13.8)  White: 439 (48.2)  Other: 84 (9.2) | Sexual minority | Illegal | Not specified | Not specified | Not specified |
| Studer *et al.* (2021) [94]  Switzerland | Cross-sectional  Online survey | N=2344  During COVID-19: 29.1 (1.3)  Not specified | Not specified | Youth/young adults/students | Illegal | Closure of borders, schools, and non-essential businesses  Physical distancing  Ban on large gatherings  Stay-at-home orders  Work from home | Not specified | Not specified |
| Sylvestre *et al.* (2022) [95]  Canada | Longitudinal  In-person and online surveys | N=972  Baseline: 20.4 (0.8)  Females: 410 (58.2%) Males: 294 (41.8%) | European Ancestry: 518 (77.7%) Other Ancestry: 149 (22.3%) | Youth/young adults/students | Legal | Closure of daycares, schools, post-secondary institutions, and non-essential businesses  Bans on large gatherings | Not specified | Not specified |
| Sznitman *et al.* (2022) [96]  Israel | Cross-sectional  Online survey | N=755  27.6 (7.0)  Males: 635 (84.2%) | Not specified | People who use cannabis | Legal for medical purpose  Illegal for recreational non-medical purposes | Quarantine  Closure of schools and universities  Travel restrictions  Restrictions on movement  Mask mandates | Recreational non-medical | Not specified |
| Tavolacci *et al.* (2021) [97]  France | Cross-sectional  Online survey | N=3671  20.9 (2.5)  Females: 2676 (72.9%) Males: 995 (27.1%) | Not specified | Youth/young adults/students | Not specified | Closure of non-essential businesses  Closure of universities  Stay-at-home orders | Not specified | Not specified |
| Terry-McElrath *et al.* (2022) [98]  USA | Repeated cross-sectional  In school paper surveys, online surveys | N=7845  Not specified (12th grade students)  Females: 48.2% Males: 40.8% Other/missing: 11.0% | Black: 11.0% Hispanic: 19.5%  White: 56.1%  Other: 13.4% | Youth/young adults/students | Not specified | Social distancing  Limitations on social gatherings and in-person school attendance. | Not specified | Not specified |
| Tham *et al.* (2022) [99]  USA and Canada | Longitudinal  Online survey | N=196  21.1 (1.6)  Males: 40 (20.4%) Females: 156 (79.6%) | White: 150 (76.5%) Hispanic: 17 (8.6%) Black: 6 (3.1%) Asian: 4 (2.1%) Multi-ethnicity/racial: 18 (9.2%) | Youth/young adults/students | Not specified | Not specified | Not specified | Not specified |
| Tholen *et al.* (2022) [100]  Belgium | Cross-sectional  Online survey | N=18346  Range = 17–24 years Females: 13709 (74.7%) Males: 4637 (25.3%) | Not specified | Youth/young adults/students | Not specified | Stay-at-home orders  Social distancing  Restriction on movements Quarantine  Remote learning | Not specified | Not specified |
| Tucker *et al.* (2020) [101]  USA | Clinical trial  In person surveys | N=90  21.9  Females: 28%  Males: 72% | Hispanic: 21%  Non-Hispanic White: 22% Non-Hispanic Black: 43%  Multiethnic/other: 13% | Youth/young adults/students | Not specified | Not specified | Not specified | Not specified |
| Turna *et al.* (2021) [102]  International study | Cross-sectional  Online survey | N=1315  42.9 (16.4)  Females: 973 (74.0%)  Males: 340 (26.0%) | Not specified | General population | Variable | Stay-at-home orders  Physical distancing | Not specified | Not specified |
| Vanderbruggen *et al.* (2020) [103]  Belgium | Cross-sectional  Online survey | N=3632  42.1 (14.6)  Females: 2541 (70.0%) Males: 1083 (29.8%) Gender-neutral: 8 (0.2%) | Not specified | General population | Not specified | Physical distancing  Stay-at-home orders | Not specified | Not specified |
| van Hooijdonk *et al.* (2022) [104]  The Netherlands | Cross-sectional  Online survey | N=9967  22.0 (2.6)  Females = 7008 (70.3%) Male 2959 (29.7%) | Not specified | Youth/young adults/students | Not specified | Social distancing  Closure of public spaces Closure of schools and universities  Online learning | Not specified | Not specified |
| Van Laar *et al.* (2020) [105]  The Netherlands | Cross-sectional  Online survey | N=1563  32.7 (12.0)  Females: 33.0% Males: 66.3% Other: 0.6% | Not specified | People who use cannabis | Illegal | Closure of non-essential businesses  Physical distancing  Ban on large gatherings | Not specified | Smoking Edibles  Vaping |
| Varin *et al.* (2021) [106]  Canada | Cross-sectional  Online survey | N=12344  18–24 years = 9.5% 25–34 years = 18.7% 35–44 years = 16.8% 45–54 years = 15.6% 55–64 years = 17.2% 65+ years = 22.2%  Females: 50.7% Males: 49.1% | Racialized group:  Yes: 24.3% No: 75.7% | General population | Not specified | Hand hygiene  Mask mandates  Closure of schools  Physical distancing | Not specified | Not specified |
| Vedelago *et al.* (2022) [107]  Canada | Cross-sectional  Online survey | N=137  31.0 (9.5)  Females: 44.5% Males: 54.7% Other: 0.7% | Caucasian/White: 73.7% East Asian/South-East Asian/Pacific Islander: 13.1% South Asian: 7.3% Hispanic/Latino: 5.8% Middle Eastern/North African/Central Asian: 4.4% Black: 2.2% Aboriginal: 1.5% Other: 2.9% | General population | Legal | Not specified | Not specified | Concentrates  Dried flower Edibles  Other |
| Vidot *et al.* (2020) [108]  USA | Cross-sectional  Online survey | N=1202  47.2 (15.2)  Females: 564 (46.9%) Males: 625 (52.0%) Transgender: 12 (1.1%) No Response: 1 (0.1%) | Non-Hispanic White: 987 (82.5%) Non-Hispanic Black: 42 (3.5%) Hispanic/Latino: 104 (8.7%) Other: 69 (5.7%) | People who use cannabis | Legal for medical use | Social distancing  Self-isolation  Lock downs  Quarantine | Medical,  Recreational non-medical | Edibles  Pills  Smoking Tinctures |
| Villanti *et al.* (2022) [109]  USA | Cohort  Online survey | N= 874  19.9 (3.5)  Males: 256 (29.4%) Females: 614 (70.6%) Cisgender: 819 (93.7%) Transgender/ don't know/ questioning: 55 (6.3%) | White: 717 (82.1%) Non-white/other race: 64 (7.3%) Hispanic: 92 (10.5%) | Youth/young adults/students | Not specified | Stay-at-home orders  Closure of schools  Remote or hybrid learning | Not specified | Not specified |
| von Soest *et al.* (2022) [110]  Norway | Repeated cross-sectional  Online survey | N=227258  15.3 (1.6)  Females: 50% | Not specified | Youth/young adults/students | Not specified | Physical distancing  Restrictions on recreational activities  Closure of schools  Online learning | Not specified | Not specified |
| Wang *et al.* (2021) [111]  USA | Longitudinal  In person and phone surveys | N=222  50.2 (11.2)  Females: 112 (50.9%) Males: 108 (49.1%) | Hispanic: 32 (14.5 %) Non-Hispanic White: 35 (15.8 %) Non-Hispanic Black: 143 (64.7 %) Non-Hispanic Other: 11 (5.0 %) | People with health/mental health comorbidities | Not specified | Not specified | Medical,  Recreational non-medical | Smoking  Vaping  Other |
| Weber *et al.* (2020) [112]  Brazil | Cross-sectional  Online survey | N=1145  37.4 (1.4)  Females: 750 (65.5%) | White (European/Western descendants): 734 (64.1%) Brown (descendant from individuals with different colors and ethnicities - Miscegenation): 332 (29.0%) Black (African/Afro-Brazilian descendants): 61 (5.3%) Yellow (Asian/Eastern descendants): 15 (1.3%) | General population | Illegal | Social distancing  Mask mandates  Hand hygiene  Restrictions on movement | Not specified | Not specified |
| Weyandt *et al.* (2020) [113]  USA | Cross-sectional  Online survey | N=302  18-24 years = .3% 25-34 years = 7.6% 35-44 years = 23.2% 45-54 years = 26.9% 55-64 years = 21.7% 65-74 years = 17.7% 75+ years = 2.4%  Females: 182 (55.8%) Males: 140 (42.9%) Transgender: 1 (.3%) Non-binary: 2 (.6%) Prefer not to say: 1 (.3%) | American Indian/Alaskan Native: 1 (.3%) Asian: 30 (8.8%) Native Hawaiian or other Pacific Islander: 1 (.3%) Black or African American: 3 (.9%) White: 264 (77.9%) Bi-/multiracial: 7 (2.1%) Latinx/Hispanic: 15 (4.4%) | Occupational | Not specified | Online learning  Limiting social contact | Not specified | Not specified |
| Xuereb *et al.* (2021) [114]  USA | Cross-sectional  Online survey | N=424  37.9 (12.3)  Females: 153 (36.1%) Males: 270 (63.7%) Other: 1 (.2%) | Ethnicity White: 307 (72.4%) Black: 71 (16.7%) Latin American: 17 (4.0%) Other: 29 (6.8%) | People with health/mental health comorbidities | Not specified | Not specified | Not specified | Not specified |
| Yousufzai *et al.* (2022) [115]  Canada | Cross-sectional  Online survey | N=312  22.8 (3.4)  Females: 189 (60.6%) Males: 123 (39.4%) | Non-white/mixed: 132 (42.3%)  White: 180 (57.7%) | Youth/young adults/students | Legal | Not specified | Not specified | Smoking  Vaping |
| Zajacova *et al.* (2020) [116]  Canada | Cross-sectional  Online survey | N=4319  25–34 years = 20%  35–44 years = 19%  45–54 years = 18%  55–64 years = 19%  65–74 years = 17% 75+ years = 7%  Females: 51% | Not specified | General population | Legal | Not specified | Not specified | Not specified |
| Doherty (2021) [117]  Australia | Cross-sectional  In-person surveys | N=446  M = 35 years  Males: 381 (85%) | Non-Indigenous: 313 (70%) | People who use substances | Illegal | Not specified | Recreational non-medical | Not specified |
| Peacock *et al.* (2020) [118]  Australia | Repeated cross-sectional  Interviews delivered via phone/videoconference | N=805  Median = 22 years  Male: 2019 = 60%, 2020 = 61% | Aboriginal and/or Torres Strait Islander: 2019 = 5%, 2020 = 4% | People who use substances | Illegal | Restrictions on gatherings Restrictions on movements | Not specified | Edibles  Smoking  Vaping |
| Bruton *et al.* (2021) [119]  Ireland | Cross-sectional  Online survey | N=696  18-21 years = 14% 22-24 years = 26% 25-30 years = 32% 31-35 years = 12% 36-45 years = 14% Over 45 = 4%  Females: 27% Males: 71% Non-binary: 1% Prefer not to say: 1% | Not specified | People who use substances | Illegal | Not specified | Recreational non-medical | Not specified |
| Johnston *et al.* (2022) [120]  USA | Repeated cross-sectional  In person and online surveys | 2020: N=11821  2021: N=32260  Not specified  Not specified | Not specified | Youth/young adults/students | Illegal | Not specified | Not specified | Edibles  Smoking  Vaping |
| Aldridge *et al.* (2021) [121]  UK | Cross-sectional  Online survey | N=2621  18-24 years = 56% 25-34 years = 25%  Males: 69% | Not specified | General population | Illegal | Not specified | Not specified | Not specified |
| Statistics Canada (2021) [122]  Canada | Cross-sectional  Online survey | Not specified  Not specified  Not specified | Not specified | General population | Not specified | Not specified | Not specified | Not specified |
| NANOS Research (2020) [123]  Canada | Cross-sectional  Telephone and online survey | N=1036  18 - 34 years = 223 35 - 54 years = 464 55+ years = 349  Females: 507 Males: 529 | Not specified | General population | Not specified | Not specified | Not specified | Not specified |
| Baillie *et al.* (2021) [124]  Australia | Cohort  Online survey | N=197  Median=27 years  Females: 97 (49.7%) Males: 91 (46.7%) Non-binary: 7 (3.6%) | Not specified | People who use substances | Illegal | Not specified | Not specified | Not specified |
| EMCDDA (2022) [125]  International study | Cross-sectional  Online survey | N=48469 18 and over  Not specified | Not specified | People who use substances | Illegal | Not specified | Not specified | Herbal cannabis |
| Peacock *et al.* (2021) [126]  Australia | Cross-sectional  Phone and face-to-face | N=884  44 (9)  Males: 59-68% | Not specified | People who use substances | Illegal | Restrictions on movement Restrictions on gatherings | Not specified | Edibles  Smoking  Vaping |
| Mental Health Commission of Canada (2021) [127]  Canada | Repeated cross-sectional  Online survey | Baseline:  N=2502  16 to 24 years = 13% 25 to 39 years = 25% 40 to 64: years = 42% 65+ years = 21% Females: 48% Males: 51% | White: 77% East/Southeast Asian: 8% Indigenous Peoples (First Nations, Inuk, Inuit, Métis): 5% South Asian: 5% Black: 2% Middle Eastern: 2% Latino: 1% Canadian / French Canadian (Non specified): 1% | General population | Not specified | Not specified | Not specified | Not specified |
| New Zealand Drug Foundation (2020) [128]  New Zealand | Cross-sectional  Online survey | N=60  Individual respondents were a range of ages  Predominantly male | Predominantly Pākehā/NZ European | People who use substances | Illegal | Not specified | Medical | Not specified |
| Statistics Canada (2020) [129]  Canada | Not specified  Online survey | Not specified  15 to 65 years and older  Not specified | Not specified | General population | Legal | Not specified | Not specified | Not specified |

**Table B: Change in cannabis use during the COVID-19 pandemic and factors associated with change in use**

| **Category** | **Author (year)** | **Outcome of interest/purpose of study** | **Tools of measurement (Self-reported)** | **Change in cannabis use** | **Factors associated with change in use** |
| --- | --- | --- | --- | --- | --- |
| 1: General population | Bonny-Noach *et al.* (2021) [11] | Substance use patterns during the COVID-19 pandemic in Israel (comparing early and later stages of pandemic) | Frequency (past month) of cannabis use comparing one to two lockdowns | Sig. 🡩 in frequency of cannabis use | Not specified |
|  | Brenneke *et al.* (2022) [12] | To (1) describe changes in days of past-week cannabis use from March 10th through November 11th, 2020 among US adults who reported cannabis use (2) characterize differences in trends of use within sociodemographic subgroups and by state cannabis policy status | Frequency (days per week) of cannabis use comparing pre-pandemic to multiple time points during the pandemic | Sig. 🡩 in frequency of cannabis use in April-May 2020, returned to levels pre-pandemic levels in June-November 2020 | Increase: gender – female, race – White, living arrangement - with partner, income – low, legal status – legal medical cannabis use (not medical and recreational)  Decrease: legal status – illegal |
|  | Brotto *et al.* (2021) [13] | Self-reports of cannabis use, impact of gender and various social variables on cannabis use | Increase, decrease, or no change in cannabis use during the pandemic | 5.9% 🡩 cannabis use | Increase: age – younger, gender – female, stress, mental health concerns – anxiety, depression, social isolation/loneliness |
|  | Das *et al.* (2022) [23] | The extent to which using substances amplify or attenuate any discovered relation between state lockdown policies and mental health symptoms | Frequency (days per week) of cannabis use comparing pre-pandemic to multiple time points during the pandemic | 🡩 in cannabis use in initial 2-3 weeks, followed by 🡫 | Not specified |
|  | El-Gabalawy and Sommer (2021) [30] | To report rates of precautions and adaptive and maladaptive health behaviors during the pandemic | Increase, decrease, or no change in cannabis use during the pandemic | 2-12% 🡩 cannabis use | Increase: age - younger |
|  | Howard *et al.* (2021) [44] | To describe self-reported increases in marijuana use during the initial stay-at-home period for the COVID-19 pandemic and determine if current employment status was associated with self-reported increases in substance use behavior | Increase, decrease, or no change in cannabis use during the pandemic | 8% 🡩 cannabis use | Increase: unemployment |
|  | MacEachern *et al.* (2021) [56] | Self-reported change in cannabis consumption during COVID-19 among the entire Canadian population, which included individuals who have never used cannabis | Increase, decrease, or no change in cannabis use during the pandemic | 5-6% 🡩 cannabis use | Increase among females: mental health concerns - depression  Increase among males: age - younger, mental health concerns - anxiety, depression  Decrease among males: parents to children |
|  | Robillard *et al.* (2021) [77] | Differences in mental health and substance use worsening in people with pre-existing mental disorders compared to those without any psychiatric history | Increase or decrease in cannabis use during the pandemic | 17-19% 🡩, 5-6% 🡫 frequency of cannabis use | Not specified |
|  | Rogers *et al.* (2020) [79] | To examine if COVID-19 worry and fear differ across three groups of substances users: abstainers, pre-COVID-19 users, and COVID-19 initiators among | Start, increase, decrease, or no change in cannabis use during the pandemic | 5% started using cannabis | Starting: mental health impact of the pandemic |
|  | Salles *et al.* (2021) [83] | To evaluate changes in the consumption of cannabis during lockdown. It also compares users and non-users of the drug in relation to: (1) sociodemographic differences; (2) emotional experiences; and (3) the information available on and degree of approval of measures introduced during the lockdown period between March and October, 2020 | Quantity (joints per week) of cannabis use comparing pre-pandemic to during pandemic | 🡩 in prevalence from 2.5% to 2.8%  Sig. 🡫 in quantity of cannabis use 49 stopped, 66 started using cannabis | Starting cannabis use: other substance use (alcohol pre lockdown), boredom, less concerned about health  Stopping cannabis use: other substance use (smoking tobacco pre lockdown), financial concerns |
|  | Somé *et al.* (2022) [91] | To assess the associations between the mental health latent classes and individuals’ cannabis use during the pandemic, and whether associations differ by sex and over time | Increase, decrease, or no change in cannabis use during the pandemic | 7% 🡩 cannabis use | Increase: mental health concerns - anxiety, depression, social isolation/loneliness |
|  | Turna *et al.* (2021) [102] | The impact of COVID-19 on the mental health and substance use of an international cohort | Change in cannabis use during the pandemic | 🡩 in cannabis use | Not specified |
|  | Vedelago *et al.* (2022) [107] | How pre-existing levels of cannabis demand related to changes in cannabis use and problems during the first 30 days of the COVID-19 state of emergency | Frequency of cannabis use comparing pre-pandemic to during the pandemic, using Daily Sessions, Frequency, Age of Onset, and Quantity of Cannabis Use Inventory | 🡩 in mean frequency of cannabis use | Not specified |
|  | Aldridge *et al.* (2021) [121] | To determine the impact that the COVID-19 pandemic, and corresponding restrictions, have had on buying illegal substances | Increase, decrease, or no change in cannabis use during the pandemic | 43% 🡩, 36% 🡪, 21% 🡫 cannabis use | Not specified |
|  | Statistics Canada (2020) [129] | Not specified | Past 3 month cannabis use comparing pre-pandemic to during the pandemic | 🡩 prevalence of cannabis use from 17% to 20% | Not specified |
|  | Ben Salah *et al.* (2022) [5] | To examine changes in substance use during the early phase of the pandemic and to identify factors related to these changes | Increase, decrease, no change, or no cannabis use comparing pre-pandemic to during the pandemic | Statistically 🡪 in cannabis use 47% 🡪, 29% 🡩, 25% 🡫 cannabis use | Increase: age – younger, stress, polysubstance use |
|  | de Quervain *et al.* (2020) [24] | To gain a better understanding of the impact of the COVID-19 pandemic on mental health | Increase, decrease or no change in cannabis use during the pandemic | 57% 🡪, 37% 🡩, 6% 🡫 cannabis use | Increase: stress, mental health concerns - anxiety, depression |
|  | Dozois (2021) [27] | Impact that the COVID-19 outbreak has had on Canadians’ levels of cannabis consumption | Increase, decrease, or no change in cannabis use during the pandemic | 48% 🡪, 29% 🡩 cannabis use | Increase: mental health concerns - anxiety, depression |
|  | Imtiaz *et al.* (2021) [46] | (1) Trends in cannabis use in the overall population, (2) patterns of and risk characteristics associated with an increase in cannabis use among those who used cannabis | Frequency (past week) of cannabis use and increase, decrease, or no change in past week cannabis use at multiple time points during the pandemic | Statistically 🡪 in prevalence/frequency of use 47-56% 🡩 among those who used cannabis | Increase: age – younger, educational level – lower, financial concerns, geographical area |
|  | Imtiaz *et al.* (2022) [47] | To characterize trends in daily cannabis use and examined risk characteristics associated with daily cannabis use during the pandemic in Canada | Frequency (past week) of cannabis use at multiple time points during the pandemic | Statistically 🡪 in cannabis use at least five days a week | No significant change based on socio-demographic factors |
|  | Knell *et al.* (2020) [50] | To describe and understand (1) how drug use changed during a 6- to 8-week period from early March to mid-April 2020; (2) who were more likely to change their health behaviors, and (3) reported reasons why participants were changing their health behaviors | Increase, decrease, or no change in cannabis use during the pandemic | 53% 🡪, 36.5% 🡩, 10% 🡫 cannabis use | Increase: mental health concerns - depression, boredom, more free time  Decrease: focus on health, increased responsibility, reduced opportunities to consume cannabis, financial concerns |
|  | Lin (2022) [53] | If health behavior changes during the COVID-19 pandemic (e.g. cannabis) are associated with the probability of having anxiety symptoms for men and women, respectively | Increase, decrease, or no change in cannabis use during the pandemic | 93% 🡪, 5% 🡩, 1% 🡫 cannabis use | Increase: mental health concerns - anxiety |
|  | Rantis *et al.* (2021) [74] | To detect changes in cannabis use in the general population, during the spring 2020 lockdown in Greece | Change in cannabis use during the pandemic | Overall, 12.7% 🡪, 8.1% 🡩, 3.7% 🡫 cannabis use 🡩 in daily use from 4% to 6% 67.4% stopped | Decrease: using with friends, at work, and at night |
|  | Rolland *et al.* (2020) [80] | To describe the containment-related changes in the respondents’ daily habits of substance use | Increase, decrease, no change, or no cannabis use during the pandemic | Overall, 2.32% 🡪, 2.05% 🡩 1.73% 🡫 average daily use, 93.91% did not use cannabis | Increase: age – younger, education level – lower, general health issues, no outdoor space in residence |
|  | Vanderbruggen *et al.* (2020) [103] | To report on changes in cannabis use in the midst of the COVID-19 lockdown in Belgium and report on the major motives for the change in consumption | Quantity (joints per day) of cannabis use comparing pre-pandemic and during the pandemic | Statistically 🡪 in number of joints per day 0.7% stopped, 0.9% started | Increase: boredom, social isolation, reward after a hard working day |
|  | Varin *et al.* (2021) [106] | To (1) estimate the self-reported change in cannabis use during the second wave of the COVID-19 pandemic, and (2) disaggregate self-reported increase in cannabis use by sociodemographic characteristics and self-reported change in mental health | Increase, decrease, or no change in cannabis use during the pandemic | 19.5% 🡪, 5.4% 🡩, 1.8% 🡫 cannabis use, 73% did not use cannabis | Increase: age – younger, gender – male, income – low, mental health – worse, non-immigrant status, geographical area |
|  | Zajacova *et al.* (2020) [116] | Changes in cannabis use; socio-demographic characteristics associated with these changes | Increase, decrease, or no change in cannabis use during the pandemic | 93% 🡪, 6% 🡩, 2% 🡫 cannabis use | Increase: age – younger, education level – lower, non-immigrant status, financial concerns  Decrease: financial concerns |
|  | Statistics Canada (2021) [122] | This release focuses on alcohol and cannabis use, the two most commonly used substances in Canada | Change in cannabis use during the pandemic | 54% 🡪, 34% 🡩 cannabis use | Increase: age – younger, stress, boredom, social isolation, access to cannabis, social acceptance of cannabis  Decrease: increased responsibilities, reduced opportunities to consume, personal choice |
|  | NANOS Research (2020) [123] | To benchmark cannabis consumption during the COVID-19 outbreak, as well as drivers of behavior | Increase, decrease, or no change in cannabis use during the pandemic | 90% 🡪, 6% 🡩, 4% 🡫 cannabis use | Increase: boredom, stress, social isolation/loneliness, disrupted daily routine, high cannabis stock  Decrease: educed opportunities to consume, personal attitudes about COVID-19, not usually using cannabis, low cannabis stock |
|  | Mental Health Commission of Canada (2021) [127] | To monitor the ongoing impacts of the COVID-19 pandemic on mental health and substance use | Increase, decrease, or no change in cannabis use at two time points during the pandemic | T1: 48% 🡪, 40% 🡩, 15% 🡫 cannabis use  T2: 43% 🡩, 42% 🡪, 15% 🡫 cannabis use | Increase: mental health and substance use concerns |
|  | Benschop *et al.* (2021) [6] | To explore and describe changing patterns in substance use | Frequency (days per week) and quantity (amount per day) cannabis consumed at two time points during the pandemic | Sig. 🡫 in prevalence  Sig. 🡩 in frequency of use  Statistically 🡪 in quantity of cannabis  33% 🡩, 17% 🡫 both frequency and quantity of cannabis use  22% 🡪 in cannabis use 11% started, 16% stopped cannabis use | Increase: stress, social isolation/loneliness, access to cannabis, coping related reasons, cannabis craving/dependence |
|  | Mravčík and Chomynová (2021) [65] | Results from an online questionnaire survey performed by the National Monitoring Centre for Drugs and Addiction among general adult population of the Czech Republic focusing on the assessment of COVID-19 on substance use and addictive behavior | Increase, decrease, or no change in cannabis use during the pandemic | Overall slight reduction, most frequent users 🡩, less frequent users 🡫 cannabis use | Not specified |
|  | Weber *et al.* (2020) [112] | To examine the frequency of use and the number of doses of psychoactive substances in the context of the Covid-19 pandemic | Frequency of cannabis use comparing pre-pandemic and during the pandemic | Sig. 🡫 in prevalence cannabis use 0.3% started, 2.6% stopped | Not specified |
| 2: Youth/ young adults/ emerging adults/ students | Bonar *et al.* (2021) [10] | Self-reported perceptions of changes in cannabis among emerging adults who regularly use cannabis. | Increase, decrease, or no change in smoking, vaporizing, dabbing, eating cannabis and using cannabidiol during the pandemic | 49% 🡩, 33% 🡪, 18% 🡫 smoking cannabis 44% 🡩, 33% 🡪, 24% 🡫 vaping cannabis 37% 🡩, 34% 🡪, 28% 🡫 dabbing cannabis  45% 🡪, 36% 🡩, 20% 🡫 eating cannabis 59% 🡪, 25% 🡩, 16% 🡫 cannabidiol | Increase: stress, social isolation, mental health concerns - anxiety, depression |
|  | Case *et al.* (2022) [17] | Qualitative study to explore how the COVID- 19 pandemic influenced marijuana vaping perceptions and behaviors among 18 to 25-year-olds, as well as to explore potential differences by gender and user type | Qualitative assessment of impact of the pandemic on cannabis use | 46% 🡩, 32% 🡫, 14% 🡪 cannabis use, 9% mixed | Increase: boredom  Decrease: reduced access to cannabis |
|  | Chaiton *et al.* (2021) [18] | To explore patterns of changes in substance use among youth and young adults | Increase or decrease in cannabis use during the pandemic | 73% 🡩 cannabis use | Not specified |
|  | Dietz *et al.* (2022) [25] | To compare the prevalence of Pharmacological neuroenhancement (PN) among university students before and during the COVID-19-pandemic. | Past year cannabis use comparing pre-pandemic to two time points during the pandemic | Prevalence initially 🡩 from 7% to 8% then 🡫 to 5% | Not specified |
|  | Dumas *et al.* (2022) [28] | To compare adolescents’ substance use patterns at the beginning of the pandemic with three different time points during the pandemic | Past three week cannabis use at multiple time points during the pandemic | Sig. 🡩 in prevalence of cannabis use | Increase: student transitioning out of high school |
|  | Gaiha *et al.* (2020) [35] | Examined whether adolescents’ and young adults’ self-reported e-cigarette use changed before and during the pandemic | Increase in quantity of cannabis in e-cigarettes during the pandemic | 7-8% 🡩 quantity of cannabis in e-cigarettes | Not specified |
|  | Gritsenko *et al.* (2021) [41] | Hypothesized COVID-19-related fear, stress, anxiety, and substance use among Russian and Belarusian university students are significantly linked to their background characteristics and country methods used to control infection | Impact of pandemic on cannabis use | 27% 🡩 cannabis use | Not specified |
|  | Mohr *et al.* (2021) [63] | Examined the psychosocial context of alcohol use at a time of rigid shelter-in-place orders in Oregon. Marijuana was considered in relation to COVID-19 and loneliness predictors to account for such use in our examination of motivated alcohol use | Increase, decrease, or no change in cannabis use during the pandemic | 36% 🡩 cannabis use | Not specified |
|  | Papp and Kouros (2021) [69] | Hypothesized that young adults would experience increased occurrence of substance use and greater increases in substance use among individuals who reported greater loneliness, financial strain, and anxiety related to health and to becoming ill at the time of COVID-19 disruptions | Frequency (past two weeks) of cannabis use comparing pre-pandemic to during the pandemic | Sig. 🡩 cannabis use | Increase: Financial concerns, cannabis craving/dependence |
|  | Potvin *et al.* (2022) [73] | To examine the contribution of the chronotype and the changes in lifestyle habits (including cannabis use) during the first wave of the COVID-19 pandemic to sleep timing in adolescents and young adults | Frequency of cannabis use comparing pre-pandemic to during the pandemic | 🡩 in mean frequency of cannabis use | Not specified |
|  | Reuter *et al.* (2021) [76] | To assess the impact COVID-related restrictions had on students’ behaviors, habits, and mental and emotional health | Frequency (past week) of cannabis use comparing pre-pandemic to during pandemic | 🡩 prevalence of cannabis use from 15% to 20%  Sig. 🡩 in mean number of days per week  🡩 every day cannabis use from 4% to 10% | Not specified |
|  | Romm *et al.* (2022) [82] | Compared changes in substance use based on individual retrospective self-report at one time-period (March-May 2020) versus prospective longitudinal self-report across 2 time-periods (Sept–Dec 2019; March–May 2020) | Increase, decrease, or no change in cannabis use during the pandemic for retrospective change and past month cannabis use comparing pre-pandemic to during pandemic for prospective change | Retrospective assessment: 54% 🡩, 47% 🡫/🡪 cannabis use  Prospective assessment: 53% 🡩, 47% 🡫/🡪 cannabis use | Not specified |
|  | Schepis *et al.* (2021) [86] | To examine differences related to university COVID-19 closure announcements in mental health and substance use in U.S. college students | Frequency (past month) of cannabis use comparing pre-pandemic to during the pandemic | Sig. 🡩 in frequency of cannabis use | Not specified |
|  | Sharma *et al.* (2020) [89] | Self-reported changes in substance use during the pandemic. Assessed differences based on demographic characteristics, self-reported anxiety, depression, loneliness, and substance use and direction of change. | Increase or decrease in cannabis use during the pandemic | 39% 🡩, 36% 🡫 cannabis use | No significant association between change and anxiety, depression, loneliness |
|  | Sylvestre *et al.* (2022) [95] | To describe change in cannabis use from before to during the COVID-19 pandemic in young adults, and to assess whether changes in substances use were heterogeneous across subgroups based on sociodemographic characteristics and a history of mental health diagnosis | Past year cannabis use comparing pre-pandemic to during the pandemic | Prevalence of cannabis use 🡩 from 18% to 23% 32% 🡩, 18% 🡫 cannabis use 17% started, 24% stopped | Increase: education level - lower, living arrangement - alone, mental health concerns  Decrease: living with children |
|  | Tucker *et al.* (2020) [101] | Surveyed 18- to 25-year-olds currently or recently homeless to understand effects of the outbreak on their substance use | Increase, decrease, or no change in cannabis use during the pandemic | 28% 🡩 cannabis use | Not specified |
|  | van Hooijdonk *et al.* (2022) [104] | To (1) investigate changes in the prevalence of weekly cannabis use before and during the first COVID-19 lockdown; (2) explore which pre-COVID-19 student-, and study-related characteristics contribute to changes in weekly cannabis use | Frequency (weekly) of cannabis use comparing pre-pandemic to during the pandemic | Sig. 🡩 in prevalence of weekly cannabis users | Increase: gender – male, living arrangement - not living with parents, education level – lower (bachelor as compared to masters), immigrant status, financial concerns, more time spent working  Decrease: age – older, more personal study time, education level – lower (bachelor as compared to masters) |
|  | Villanti *et al.* (2022) [109] | To explore the relationships between COVID-related distress, mental health symptoms, and substance use | Increase, decrease, or no change in cannabis use during the pandemic | 50% 🡩, 33% 🡪, 15% 🡫 cannabis use | Not specified |
|  | Busse *et al.* (2021) [14] | Is there any change of engagement in Health Risk Behaviors (HRB) in German university students during the COVID-19 pandemic? What characteristics are associated with a change of engagement in HRB? | Frequency of cannabis use comparing pre-pandemic to during the pandemic | 93.3% 🡪, 3.9% 🡫, 2.8% 🡩 frequency of cannabis use | Increase: mental health concerns - depression  Decrease: age – younger, gender - female |
|  | Clendennen *et al.* (2021) [19] | To examine the prevalence of self- reported COVID-19–related changes in past 30-day marijuana use behaviors | Increase, decrease, or no change in cannabis use during the pandemic | 41% 🡪, 37% 🡩, 22% 🡫 cannabis use | Increase: age – older, race/ethnicity – non-White, substance use concerns, stress |
|  | Firkey *et al.* (2020) [33] | To assess substance use since the onset of COVID-19 | Increase, decrease, or no change in cannabis use during the pandemic | 57.5% 🡪, 26% 🡫, 15% 🡩 cannabis use | Decrease: gender - male |
|  | Graupensperger *et al.* (2021) [40] | To estimate intra-individual changes in marijuana use. Whether changes in use differed by theoretically important demographic characteristics. | Frequency (highs per week/use days per week) of cannabis use comparing pre-pandemic to during the pandemic | Statistically 🡪 in highs per week/use days per week 21% 🡩, 14.5% 🡫 hours high per week 15% 🡩, 9% 🡫 cannabis use days per week | No significant difference based on legality of cannabis |
|  | Jodczyk *et al.* (2022) [49] | To evaluate the impact of the COVID-19 pandemic on usage of marijuana and products containing tetrahydrocannabinol (THC) | Increase, decrease, or no change in cannabis use during the pandemic | 80% 🡪, 7% 🡩, 13% 🡫 cannabis use | No significant change based on socio-demographic factors, being a medical student, poorer mental health |
|  | Miech *et al.* (2021) [61] | Changes in adolescent substance use during the COVID-19 pandemic | Past month frequency of cannabis use comparing pre-pandemic to during the pandemic | Statistically 🡪 in past 30 days | Not specified |
|  | Nguyen *et al.* (2021) [66] | Self-reported changes in cannabis vaping during the early stage of the pandemic among 1,553 adolescents and young adults who reported ever vaping cannabis. Also examined factors associated with any increased cannabis vaping | Increased cannabis vaping, decreased cannabis/nicotine vaping, no change in cannabis/nicotine vaping, or stopping cannabis/nicotine vaping during the pandemic | 42% 🡪 cannabis/nicotine vaping  18% 🡫 cannabis/nicotine vaping  7% 🡩 cannabis vaping 19% stopped cannabis/nicotine vaping | Increase: gender – female, personal attitudes - safety of vaping cannabis vs. smoking cigarettes, cannabis craving/dependence  Decrease: personal attitudes - cannabis vaping risks |
|  | Pocuca *et al.* (2022) [72] | To examine changes in cannabis use (CU) among emerging adults from prepandemic (21 years) to during COVID-19 (22 years), whether prepandemic CU, COVID-19-related factors, and preexisting vulnerabilities moderated change in CU during COVID-19 | Frequency of cannabis use comparing pre-pandemic to during the pandemic | Statistically 🡪 in frequency  Statistically 🡪 in less than monthly use Sig. 🡫 in more than monthly use | Increase: financial concerns |
|  | Romm *et al.* (2022) [81] | (i) changes in marijuana use, from pre COVID-19 to during COVID-19, (2) depressive symptoms and Adverse Childhood Experiences as risk factors for changes in use frequency of each substance; and (3) the extent to which resilience moderates the relationship between depressive symptoms or ACES and changes in substance use | Frequency (past month) cannabis use comparing pre-pandemic to during the pandemic | Among cannabis users, 52% 🡪, 27% 🡩, 21% 🡫 in frequency of cannabis use  Among frequent cannabis users, 53% 🡪, 31% 🡩, 15% 🡫 | Increase: loss of employment, living arrangement - not living with parents, less frequent cannabis use pre lockdown, lower dispositional resilience |
|  | Salmon *et al.* (2022) [84] | To examine self-reported changes in consumption of cannabis due to the COVID-19 pandemic; to determine if differences exist based on respondent sex, age group, or household income after adjusting for sociodemographic characteristics and pre-pandemic self-rated mental health | Increase, decrease, or no change in cannabis use during the pandemic | 44% 🡪, 35% 🡩, 21% 🡫 cannabis use | No significant change based on socio-demographic factors |
|  | Shapiro *et al.* (2022) [88] | To investigate drug use, among adolescents in Israel during the COVID-19 pandemic and whether the pandemic influenced the prevalence of these risky behaviors | Lifetime, past year, past month frequency of cannabis use | 50% 🡪, 16% 🡩, 5% 🡫 cannabis use 15% started, 15% stopped | Not specified |
|  | Studer *et al.* (2021) [94] | To assess changes in illegal cannabis use during the COVID-19 crisis | Past year cannabis use comparing pre-pandemic to during the pandemic | Statistically 🡪 cannabis use | Increase: other activities - watching TV |
|  | Tham *et al.* (2022) [99] | Compared substance use behaviors 1 year before the pandemic, to 6 months after its onset in young adults with chronic pain | Past 3 month frequency of cannabis use comparing pre-pandemic to during pandemic using the Alcohol, Smoking and Substance Involvement Screening Test (ASSIST) | Statistically 🡪 in prevalence of cannabis use | Not specified |
|  | Yousufzai *et al.* (2022) [115] | To examine if there was a change in self-reported frequency of inhaled routes of cannabis consumption (i.e., smoking and vaping) following the COVID-19 pandemic | Frequency of cannabis use comparing pre-pandemic to during the pandemic | Statistically 🡪 in mean frequency of cannabis smoking and vaping | Increase: disrupted daily routine/more free time, stress, mental health concerns - anxiety  Decrease: reduced opportunities to consume, personal attitudes about COVID-19 risk/harm |
|  | Bartel *et al.* (2020) [3] | Hypothesized both self-isolation and coping with depression motives would predict cannabis use levels during the pandemic, after controlling pre-pandemic levels of cannabis use | Past month frequency and quantity of cannabis use comparing pre-pandemic to during pandemic, using the Daily Sessions, Frequency, Age of Onset, and Quantity of Cannabis Use Inventory | 🡫 in mean quantity and frequency of cannabis | Not specified |
|  | Hicks *et al.* (2022) [42] | To examine factors (e.g., mental health, coping behaviours, environmental aspects, social groups) that may influence substance use with data gathered from a longitudinal study of college students. | Past year and current frequency of cannabis use comparing pre-pandemic to during the pandemic using the Cannabis Use Disorder Identification Test-Revised | No cannabis use: 🡩 from 65% to 77% Monthly use or less: 🡫 from 18% to 7% 2 to 4 times a month: 🡫 from 5% to 4%  2 to 3 times a week: 🡫 from 5% to 3% 4 or more times a week: 🡩 from 7% to 9%  20% 🡫, 11% 🡩 frequency of cannabis use | Increase: identifying as a sexual minority, dispositional impulsivity, mental health concerns - PTSD, justice system involvement, support disruption  Decrease: Sleep quality satisfaction, change in living arrangement, stressful friendships, positive life changes due to COVID-19, discrimination |
|  | Lukács (2021) [55] | Hypothesized that university students would increase the amount of drug consumed compared to the amounts reported retrospectively prior to the outbreak of the virus | Frequency of cannabis use comparing pre-pandemic to during the pandemic | Sig. 🡫 in frequency of cannabis use | Not specified |
|  | Merrill *et al.* (2022) [59] | Hypothesized that, on average, students would decrease cannabis use from pre-closure to post-closure-1. Also explored changes in specific formulations and characterized self-reported reasons for perceived increases and decreases in cannabis use frequency to further contextualize changes in cannabis use. | Frequency (days per week) of cannabis use, any use, leaf, concentrates, and edible cannabis use comparing pre-pandemic to two time points during pandemic | Sig. 🡫 in any use, leaf, concentrates, and edibles from pre-pandemic to first time point during pandemic  Statistically 🡪 frequency of cannabis use | Increase: boredom, more free time, coping related reasons  Decrease: reduce opportunities to consume, cannabis product - using leaf, edibles, concentrate, edibles, living with parents |
|  | Tavolacci *et al.* (2021) [97] | To assess changes in health behaviors (cannabis use) during the COVID-19 outbreak and lockdown and to identify factors associated with favorable and unfavorable changes in health behaviors | During the month before the pandemic and during the pandemic (past week) cannabis use | Sig. 🡫 in prevalence of cannabis use | Increase: Gender – male, return to living with parents, staying alone, depression  Decrease: Gender – male, mental health concerns - depression |
|  | Terry-McElrath *et al.* (2022) [98] | To estimate solitary marijuana use prevalence across 2015–2021 and trends and/or deviations associated with the pandemic; examine associations between reasons for use and solitary use | Past year and solitary cannabis use comparing pre-pandemic to during the pandemic | Overall, 🡫 in cannabis use  🡩 in solitary cannabis use | No significant association with socio-demographic factors |
|  | Tholen *et al.* (2022) [100] | To what extent are quarantine stressors and psychosocial distress associated with Belgian students’ self-reported substance use before and during the first wave of the COVID-19 pandemic | During the month before the pandemic and during the pandemic (past week) cannabis use | 59% 🡫, 23% 🡩, 19% 🡪 cannabis use | Increase: mental health concerns – depression, living arrangement – not living with parents  Decrease: living with parents |
|  | von Soest *et al.* (2022) [110] | What are the psychosocial changes for adolescents one year after the onset of the pandemic? Are the changes disproportionately large for adolescents from disadvantaged backgrounds? Do the changes vary according to geographical variations in infection rates and restrictions? | Past year cannabis use comparing pre-pandemic to during the pandemic | Sig. 🡫 in cannabis use | Decrease: Perceived family poverty |
|  | Johnston *et al.* (2022) [120] | Ongoing collection of valid and reliable data about substance use among 8, 10, 12 graders | Lifetime, past year, and past month cannabis use comparing pre-pandemic to during the pandemic | Sig. 🡫 in lifetime, annual and past month prevalence, daily cannabis use and cannabis vaping | Not specified |
| 3: People who use cannabis | Cousijn *et al.* (2021) [21] | To (i) investigate if lockdown was associated with change in cannabis use (ii) investigate if pre- to post-lockdown change in cannabis use and CUD symptom severity are related to change in cannabis use motives, mental wellbeing, quality of social relationships and job status | Frequency (days per week) and quantity (grams per month) cannabis use comparing pre-pandemic to during the pandemic | Sig. 🡩 in frequency and quantity of cannabis use | Increase: social isolation/loneliness, lower pre lockdown cannabis use, social/expansion motives |
|  | Fedorova *et al.* (2021) [31] | (1) the extent to which patterns of cannabis use changed after safer-at-home order went into effect in California and (2) reasons and contexts of changes in cannabis use within a sample of medical and recreational younger adult cannabis users | Frequency and quantity of cannabis use comparing pre-pandemic to during the pandemic and qualitative assessment of impact of the pandemic on cannabis use | Sig. 🡩 in cannabis use days, hits per day, and amount among those who increased  29% 🡩 cannabis use | Increase: race/ethnicity - Black/African Americans, loss of employment, positive life changes due to COVID-19 |
|  | Gattamorta *et al.* (2021) [36] | To examine the mental health burdens and related coping mechanism of LGBTQ cannabis users amid the COVID-19 pandemic | Impact of pandemic on cannabis use using the COVID-19 Cannabis Health Questionnaire (CCHQ) | 39-44% 🡩 quantity of cannabis use | Increase: identify as LGBTQ+ |
|  | Lake *et al.* (2022) [51] | To examine changes in the prevalence, frequency, and inhalation of medical cannabis during COVID-19 for a range of conditions and symptoms commonly self-managed with cannabis. To identify factors associated with shifts in cannabis use for selective therapeutic indications during COVID-19 | Frequency of medical cannabis use, and inhalation versus non-inhalation of cannabis, comparing pre-pandemic to during pandemic | Sig. 🡩 in frequency  29% 🡩, 18% 🡫 frequency of medical cannabis Prevalence of occasional users 🡫 from 62% to 56% Prevalence of weekly users 🡩 from 21% to 23% Prevalence of daily users 🡩 from 16% to 21%  Statistically 🡪 in inhalation | Increase: gender – female, more free time, legal status – legal  Decrease: age – older, risk of COVID-19 infection, geographical area, baseline cannabis use - higher, cost of cannabis |
|  | Lewis and Sznitman (2022) [52] | Whether higher frequency of media use for COVID-19 content was associated with increased incidence of harmful cannabis use patterns through maladaptive coping strategies, specifically, increased cannabis use, use when alone, and use before midday | Increase in cannabis use, increase in frequency of cannabis use, and increase in frequency of cannabis use before noon, during the pandemic | 41% 🡩 cannabis use 40% 🡩 frequency of cannabis use 34% 🡩 cannabis use before noon | Increase: coping related reasons, daily cannabis use, media use |
|  | Mezaache *et al.* (2022) [60] | To investigate the impact of the first COVID-19 lockdown on daily cannabis users—for self-medication—in France. To describe changes in their cannabis use, and to assess correlates associated with these changes | Frequency (daily) of cannabis use comparing pre-pandemic to during pandemic | 36% 🡩, 29% 🡪, 29% 🡫 cannabis use 7.3% stopped | Increase: age – younger, gender – female, education level – higher, students, working from home, loss of employment, less likely to use cannabis to self-medicate, high cannabis stock, other substance use, COVID-19 symptoms, mental health concerns – depression, sleep quality dissatisfaction  Decrease/stopping: age – younger, gender – males, education level – higher, students, rural residence, living arrangement – living with a partner, no outdoor space in residence, less likely to use cannabis to self-medicate, high cannabis stock, other substance use, mental health concerns – anxiety, depression, sleep quality dissatisfaction , health concerns – pain, dyspnea |
|  | Sznitman *et al.* (2022) [96] | To test the following hypotheses: experiencing primary and secondary COVID-19 stressors will be associated with reported increase in cannabis use (H1), the association between the COVID-19 stressors and increased cannabis use is mediated by coping motives (H2), and the direct and indirect effects of primary and secondary COVID-19 stressors on increase in cannabis use differ in magnitude and are moderated by the stage of lockdown measures (H3) | Increase in cannabis use during the pandemic | Sig. 🡩 in prevalence of use | Increase: coping motives, financial concerns, social isolation |
|  | Assaf *et al.* (2022) [2] | To assess changes in patterns of both self-reported medical and non-medical cannabis use during the pandemic across a general group of those who use cannabis and to identify variables associated with changes in use | Increase, decrease, or no change in medical, non-medical, and methods of cannabis use comparing pre-pandemic to during the pandemic | Non medical cannabis Once or twice a week: 51% 🡩, 45% 🡪, 4% 🡫 Monthly use: 48% 🡪, 32% 🡩, 20% 🡫 Weekly use: 62% 🡪, 21% 🡫, 16% 🡩  Daily or almost daily use: 50% 🡪, 30% 🡫, 20% 🡩   Medical Cannabis Once or twice use: 55% 🡪, 43% 🡩, 3% 🡫 Monthly use: 50% 🡪, 28% 🡫, 22% 🡩 Weekly use: 62% 🡪, 21% 🡫, 17% 🡩 Daily or almost daily use: 41% 🡪, 31% 🡩, 28% 🡫  🡫 inhalation and 🡩 oral cannabis use | Increase: gender – female, legal status – legal, less than or equal to monthly and daily cannabis use before the pandemic |
|  | Boehnke *et al.* (2021) [9] | How people using cannabis medically were affected by COVID-19, with specific focus on cannabis access and use of other medications and substances (e.g., alcohol, tobacco). | Increase, decrease, or no change in frequency of cannabis use since the onset of the pandemic | 40% 🡪, 35% 🡩, 25% 🡫 frequency of cannabis use | Increase: mental health impact of the pandemic, boredom, increased symptom burden  Decrease: legal status – illegal, other substance use, reduced access to cannabis, mental health impact of the pandemic, decreased responsibilities |
|  | Miller *et al.* (2022) [62] | What impact has the coronavirus pandemic had on cannabis use among recent cannabis users? | Qualitative assessment of impact of the pandemic on cannabis use | Many 🡪, some 🡩, some 🡫 | Increase: working from home, stress  Decrease: low cannabis stock, firmer boundaries on use, focus on health |
|  | Rodriguez *et al.* (2022) [78] | Examine the behavioral impact of the COVID-19 pandemic on cancer survivors who endorse cannabis compared to adults without a history of cancer | Increase, decrease, or no change in quantity of cannabis use during the pandemic | 60% 🡪, 31% 🡩, 9% 🡫 quantity of cannabis use | Increase: mental health concerns - anxiety, depression |
|  | Van Laar *et al.* (2020) [105] | Changes in cannabis use in a non-probability sample of cannabis users in the Netherlands during the early lockdown period | Increase, decrease, or no change in cannabis use during the pandemic, frequency and quantity of cannabis use comparing pre-pandemic to during the pandemic | 49% 🡪, 41% 🡩, 7% 🡫 in frequency of use  54% 🡪, 39% 🡩, 6% 🡫 quantity of use  Sig. 🡩 in average number of joints in total sample 3% stopped | Increase: age – younger, gender – female, boredom  Stopping/decrease: reduced opportunities to consume cannabis, general health and mental health concerns |
|  | Vidot *et al.* (2020) [108] | Self-reported behavioral changes among adult medical cannabis users since COVID-19 was declared a pandemic | Increase, decrease, or no change in cannabis use during the pandemic, using the COVID-19 Cannabis Health Questionnaire | 48% 🡪, 38% 🡩, 9% 🡫, cannabis use | Increase: mental health concerns |
|  | Fernández-Artamendi *et al.* (2021) [32] | To evaluate changes in the pattern of cannabis use during the confinement associated with the state of alarm in Spain | Frequency (past week) of cannabis use comparing pre-pandemic to during the pandemic | Sig. 🡫 in frequency of cannabis use | Not specified |
| 4a: People with health/mental health comorbidities | Armour *et al.* (2022) [1] | To investigate the patterns of cannabis use worldwide in self-management of endometriosis related pain during the early COVID-19 pandemic and to explore if cannabis consumption had changed because of potential restrictions, barriers to health care, or health-related concerns. | Increase, decrease, or no change in quantity and methods of cannabis use since the onset of pandemic | 56.9% 🡩, 32.2% 🡪 quantity of cannabis use  39.5% 🡫 inhalation, 40% 🡩 edibles, 25.2% 🡩 oils | Starting: mental health concerns - anxiety, stress, reduced access to healthcare, amplification of endometriosis symptoms  Decrease: reduced access to cannabis, cannabis side effects, cost |
|  | Currie (2021) [22] | Examine gender and socioeconomic differences in pandemic-related PTSD symptoms and substance use among adults | Increase, decrease, or no change in past month cannabis use during the pandemic | 2-4% 🡩 cannabis use | Increase: gender – males |
|  | Glazer and Vallis (2022) [38] | Impact of the pandemic was reported on specific weight management strategies including substance use | Extent of change in cannabis use during the pandemic | 17% 🡩 cannabis use | Increase: Obesity |
|  | Wang *et al.* (2021) [111] | To: 1) investigate the changes in marijuana use frequency and quantity among people living with HIV during the first year of the COVID-19 pandemic; 2) examine the associations between changes in marijuana use and mental health as well as perceived risks/benefits of marijuana use during the pandemic | Past month frequency and quantity cannabis use comparing pre-pandemic to during pandemic | 29% 🡩, 16% 🡫 frequency or quantity of cannabis use  3% stopped, 0.9% started cannabis use | Increase: baseline cannabis use – higher, mental health concerns – PTSD, mental health concerns, perceived that cannabis would not increase risk of COVID-19  Decrease: baseline cannabis use – higher |
|  | Xuereb *et al.* (2021) [114] | Changes in use of legal as well as illegal substances and other putative behavioral addictions during COVID-19 | Increase, decrease, or no change in cannabis use during the pandemic | Sig. 🡩 based on mean change of cannabis use | Increase: other activities - online gambling, social casino gambling, other substance use |
|  | Baum *et al.* (2022) [4] | Examined psychological resilience and anxiety in relation to substance use patterns during the pandemic among people living with and without HIV | Past month use of cannabis at two time points during the pandemic | Statistically 🡪 in cannabis use | Decrease: HIV negative status as compared to HIV positive status |
|  | Camacho-Rivera *et al.* (2021) [15] | To identify changes to cannabis use, and to describe differences in cannabis behaviors by cancer status among an age-matched sample of cannabis users from the COVID-19 cannabis health study | Increased, decrease, or no change in quantity of cannabis use during the pandemic | 59% 🡪, 29% 🡩, 12% 🡫 in quantity of cannabis use | No significant difference based on cancer survivors status, demographic characteristics etc. |
|  | Donovan and Portman (2021) [26] | Examined the effect of the pandemic on cannabis use in cancer patients | Increase, decrease, or no change in cannabis use during the pandemic | 81% 🡪, 11.5% 🡫, 8% 🡩 in quantity of cannabis use | Not specified |
|  | Hochstatter *et al.* (2021) [43] | To determine whether substance use, including marijuana have increased during the COVID-19 pandemic among PLWH and substance user disorder | Past week cannabis use comparing pre-pandemic to during the pandemic | Statistically 🡪 in prevalence of cannabis use | Not specified |
|  | Meanley *et al.* (2022) [58] | To compare the short-term trajectories of marijuana use spanning pre-COVID-19 pandemic to early pandemic time points among a prospective cohort of PLWH | Frequency of cannabis use comparing pre-pandemic to during the pandemic | Statistically 🡪 in cannabis use among non-frequent or daily users | Not specified |
| 4b: People who use substances | Carlyle *et al.* (2021) [16] | To: (i) explore the impact of COVID-19 on patterns of alcohol and other drug (AOD) use among people who had sought treatment for a substance use disorder in Australia during the pandemic; (ii) identify whether mental health variables such as depression, anxiety, trauma and resilience were associated with changes in AOD use and (iii) evaluate changes in contextual factors associated with AOD use | Increase, decrease, or no change in frequency and quantity of cannabis use during the pandemic | Sig. 🡩 quantity of cannabis use 46% 🡩, 25% 🡫, 31% 🡪 frequency of cannabis use 39% 🡩, 30% 🡫, 32% 🡪 quantity of cannabis use | Increase: difficulty accessing cannabis  Decrease: mental health concerns - anxiety, dispositional resilience |
|  | Lintzeris *et al.* (2021) [54] | Examine changes in patient outcomes (including substance use) in the 6 months following the implementation of opioid agonist treatment changes in response to COVID-19 | Frequency of cannabis use comparing pre-pandemic to during the pandemic | Sig. 🡩 in past month use, and frequency of use | Not specified |
|  | Manthey *et al.* (2021) [57] | Self-reported changes of cannabis use based on survey data collected between April and July 2020 | Increase, decrease, or no change in cannabis use during the pandemic | Sig. 🡩 in cannabis use | Not specified |
|  | Palamar *et al.* (2020) [68] | To determine whether frequency or amount of use of various drugs changed during COVID-19 social distancing | Increase, decrease, or no change in frequency and quantity of cannabis during the pandemic | 35% 🡩, 33% 🡪, 32% 🡫 in frequency 52% 🡪, 19% 🡩, 29% 🡫 in quantity | Decrease: education level - higher |
|  | Doherty (2021) [117] | Explored changes in the demand for and supply of cannabis in Perth, Brisbane, Adelaide and Sydney during the COVID-19 pandemic | Past month frequency and quantity of cannabis use comparing pre-pandemic to during the pandemic | Sig. 🡩 in frequency of cannabis use Statistically 🡪 in quantity of cannabis use | Increase frequency/quantity: change in their employment, financial or living situation, mental health impact of the pandemic, coping related reasons |
|  | Peacock *et al.* (2020) [118] | EDRS is designed to be sensitive to emerging trends about substance use, providing data in a timely manner rather than describing issues in extensive detail | Past month cannabis use, frequency, quantity, modes of consumption comparing pre-pandemic to during the pandemic | Sig. 🡩 in prevalence of cannabis use Sig. 🡩 in swallowing and vaping cannabis Statistically 🡪 in quantity of cannabis use  Sig. 🡫 in daily cannabis users 41% 🡪, 39% 🡩, 11% 🡫 cannabis use 6% stopped, 3% started | Increase: boredom, more free time, mental health concerns – anxiety, depression |
|  | Bruton *et al.* (2021) [119] | To gather information on how patterns of drug use have changed in Europe during the COVID-19 pandemic | Past year, past month cannabis use comparing pre-pandemic to during the pandemic, and increase, decrease, or no change in frequency and quantity of cannabis use during the pandemic | 32% 🡩, 17% 🡫 frequency of cannabis use 13% 🡩 quantity, 5% 🡫 quantity of cannabis use 30% 🡪 cannabis use 12% stopped, 2% started | Not specified |
|  | Baillie *et al.* (2021) [124] | ADAPT Study explores the short and long-term impact of the COVID-19 pandemic on the experiences of Australians who use illicit drugs | Increase, decrease, or no change in cannabis use at multiple time points during the pandemic | Wave 1: 🡩 56%, 32% 🡪, 🡫 12%  Wave 2: 🡩 46%, 36% 🡪, 🡫 18% Wave 3: 37% 🡪, 🡫 33%, 🡩 30% Wave 4: 🡫 35%, 🡩 33% , 32% 🡪 cannabis use | Not specified |
|  | EMCDDA (2022) [125] | To improve understanding of patterns of drug use in Europe and help shape future drug policies and interventions | Change in cannabis use during the pandemic | 32% 🡩 cannabis use | Not specified |
|  | New Zealand Drug Foundation (2020) [128] | Pulse surveys quickly capture what changes were ‘top-of-mind’ for addiction services and people using alcohol and other drugs during Alert Level 4 of the COVID-19 response | Change in cannabis use during the pandemic | Most participants 🡩 (14), some 🡫 (4) cannabis use | Increase: mental health concerns - anxiety, boredom  Decrease: reduced access to cannabis, reduced opportunities to consume |
|  | Gaume *et al.* (2021) [37] | To collect first-hand observations by substance users purchasing drugs in the illegal market | Past week cannabis use, frequency, and quantity at two time points during the pandemic. Qualitative assessment of the impact of the pandemic on cannabis use | Statistically 🡪 in cannabis use, frequency, or quantity Prevalence 🡫 from 51% in wave 1 to 35% in wave 2 | Increase: other substance use  Decrease: social isolation, financial concerns |
|  | Scherbaum *et al.* (2021) [87] | To collect data from users of illicit drugs, regarding the availability of their preferred substances within the context of the COVID-19 pandemic | Change in frequency of cannabis use during the pandemic | 84% 🡪 in frequency of cannabis use | Not specified |
|  | Blithikioti *et al.* (2021) [7] | To analyze lockdown’s effects on substance use and mental health of patients under treatment for a substance use disorder diagnosis | Selected questions from the ASSIST to measure frequency of cannabis use comparing pre-pandemic to during the pandemic | Sig. 🡫 in frequency of use.  10% 🡫, 1% 🡩 frequency of cannabis use | Not specified |
|  | Otiashvili *et al.* (2022) [67] | To examine changes in drug taking behaviors over the first six months of the pandemic through collecting prospective longitudinal data from the cohorts of people who use drugs regularly in Georgia | Frequency (past two weeks) of cannabis use at multiple time points during the pandemic. Qualitative assessment of impact of pandemic on cannabis use. | Sig. 🡫 in frequency of cannabis use | Decrease: reduced access to cannabis, reduced opportunities to consume/reduced social contact, mental health impact of the pandemic |
|  | Pavarin *et al.* (2022) [70] | Changes in habits and in consumption styles in the months during the lockdown due to COVID-19 | Frequency (past month) and quantity (per day) of cannabis use comparing pre-pandemic to during the pandemic | Sig. 🡫 in prevalence of cannabis use | Not specified |
|  | Sande *et al.* (2021) [85] | Changes in the characteristics of drug use among people who used drugs more often and were going out more frequently before the COVID-19 epidemic | Cannabis use comparing pre-pandemic to during the pandemic and increase, decrease or no change in frequency and quantity of cannabis use during the pandemic | Overall prevalence 🡫 from 75% to 60%  43.5% 🡩, 38% 🡪, 19% 🡫 frequency of cannabis use 41% 🡩 quantity of cannabis use | Not specified |
|  | Peacock *et al.* (2021) [126] | IDRS provides a coordinated approach to monitoring the use, market features, and harms of illicit drugs, is sensitive to emerging trends, providing data in a timely manner, rather than describing issues in extensive detail | Past 6 month cannabis use, frequency, quantity, and modes of consumption comparing pre-pandemic to during the pandemic and change in cannabis use the pandemic | Sig. 🡫 in prevalence of recent use Statistically 🡪 in median frequency, quantity, or route of administration 64% 🡪, 25% 🡩, 9% 🡫 cannabis use 3% stopped, 5% started | Increase: boredom, mental health concerns - depression/anxiety, more free time |
| 5: Occupational | Conroy *et al.* (2021) [20] | Differences between substance use in health care workers who reported in person compared with these differences among those who worked primarily from home | Frequency of cannabis use comparing pre-pandemic to during the pandemic | Sig. 🡩 in frequency of cannabis use | Not specified |
|  | Imboden *et al.* (2021) [45] | Detect changes in cannabis use in elite athletes and bodybuilders during the first COVID-19 lockdown in Switzerland | Cannabis use and past month frequency comparing pre-pandemic to during the pandemic | Sig. 🡩 in frequency among athletes who continued to use cannabis | Increase type of athlete: bodybuilder vs. elite athlete |
|  | Weyandt *et al.* (2020) [113] | To investigate the relationship among anxiety, depression, impulsivity, health behaviors, and mindfulness among faculty members during the pandemic | Change in cannabis use during the pandemic | 2.7% 🡩 cannabis use | Increase: race/ethnicity - non-Latinx/Hispanic participants, mental health concerns |
|  | Pedersen *et al.* (2021) [71] | Veterans’ reactions to COVID-19 and substance use patterns, expected cannabis use to either stay approximately the same or increase due to stress related to the pandemic | Lifetime and past month frequency of cannabis use comparing pre-pandemic to during the pandemic | Sig. 🡩 in lifetime use  Statistically 🡪 in days of cannabis use 14% started using cannabis | Not specified |
|  | Mora *et al.* (2020) [64] | To describe the impact of the pandemic as reported by 1115 farmworkers in Monterey County in a cross-sectional study | Increase or no increase in cannabis use during the pandemic | 99.3% 🡪, 0.7% 🡩 cannabis use | Not specified |
|  | Reilly *et al.* (2022) [75] | To investigate the impact of the COVID-19 pandemic on United States veterans who self-reported problematic drug use | Increase, decrease, or no change in cannabis use during the pandemic | 53% 🡪, 35% 🡩, 13% 🡫 cannabis use | Not specified |
|  | Fitzke *et al.* (2021) [34] | To investigate the association between pre‐existing depression and substance use behaviors during the pandemic | Past month cannabis use comparing pre-pandemic to during the pandemic | 🡫 prevalence of cannabis use from 17% to 15% | Not specified |
| 6: Sexual minority | Bochicchio *et al.* (2021) [8] | How do Sexual Minority Women describe their experiences and perceptions of alcohol and marijuana use in the context of the COVID-19 pandemic and related mitigation efforts? | Qualitative assessment of impact of the pandemic on cannabis use | Many 🡩 or initiated, some 🡫 | Starting/increase: access to cannabis, legal status – legal, stress, boredom, disrupted daily routine  Decrease: reduced social contacts |
|  | Dyar *et al.* (2021) [29] | To further our understanding of risk factors for increases in substance use and consequences during the COVID-19 pandemic among a high risk population sexual and gender minority (SGM) | Change in frequency and quantity of cannabis use during the pandemic | 67% 🡩 frequency, 54% 🡩 quantity, 54% 🡩 both frequency and quantity | Increase: mental health concerns – anxiety, depression, using alone or romantic partners  Decrease: using with friends |
|  | Goodyear *et al.* (2021) [39] | To describe the extent of increased cannabis use among LGBTQ2+ adults in Canada during the COVID-19 pandemic, and then to examine associations between increased cannabis use and selected adverse mental health outcomes | Increase, decrease, or no change in cannabis use during the pandemic | 🡩 in increased cannabis use from 17% in round 1 to 24% in round 2, 🡫 in round 3 to 12% | Increase: mental health concerns - suicidality |
|  | Slemon *et al.* (2022) [90] | To compare SGM mental health and substance use impacts of the COVID-19 pandemic to non-SGM peers to identify risk factors for adverse mental health and substance use outcomes among SGMs | Increase, decrease or no change in cannabis use during the pandemic | Prevalence 🡩 from 17% to 21% in SGM participants and from 5% to 6% in non SGM participants | Increase: mental health concerns |
|  | Janulis *et al.* (2021) [48] | To examine changes in both prevalence and frequency of drug use among young men who have sex with men and young transgender women during the pandemic | Past month frequency of cannabis use comparing pre-pandemic to during the pandemic | Statistically 🡪 in prevalence | Not specified |
|  | Somé *et al.* (2022) [92] | To assess differences between transgender and cisgender Canadian  adults in their respective cannabis use during the COVID-19 pandemic | Increase, decrease or no change in cannabis use during the pandemic | Transgender or gender diverse individuals: 61% 🡪, 33% 🡩, 5% 🡫 cannabis use Cisgender individuals: 89% 🡪, 7% 🡩, 4% 🡫 cannabis use | Increase: transgender participants |
|  | Starks *et al.* (2020) [93] | Drug use and sexual behaviors among sexual minority men pre-COVID-19 and during the epidemic | Cannabis use comparing pre-pandemic to during the pandemic | Sig. 🡫 in use | Not specified |

Note: Sig. = Significant, 🡩 = Increase, 🡪 = No change, 🡫 = Decrease

**References**

1. Armour M, Sinclair J, Cheng J, Davis P, Hameed A, Meegahapola H, et al. Endometriosis and cannabis consumption during the covid-19 pandemic: an international cross-sectional survey. Cannabis and Cannabinoid Research. 2022.

2. Assaf RD, Gorbach PM, Cooper ZD. Changes in medical and non-medical cannabis use among United States adults before and during the COVID-19 pandemic. The American Journal of Drug and Alcohol Abuse. 2022:1-7.

3. Bartel S, Sherry S, Stewart S. Self-isolation: A significant contributor to cannabis use during the COVID-19 pandemic. Substance Abuse. 2020;41(4):409-12.

4. Baum MK, Tamargo JA, Diaz-Martinez J, Delgado-Enciso I, Meade CS, Kirk GD, et al. HIV, psychological resilience, and substance misuse during the COVID-19 pandemic: A multi-cohort study. Drug and Alcohol Dependence. 2022;231:109230.

5. Ben Salah A, DeAngelis BN, Morales D, Bongard S, Leufen L, Johnson R, et al. A multinational study of psychosocial stressors and symptoms associated with increased substance use during the early wave of the COVID-19 Pandemic: The role of polysubstance use. Cogent Psychology. 2022;9(1):2054162.

6. Benschop A, Van Bakkum F, Noijen J. Changing patterns of substance use during the coronavirus pandemic: Self-reported use of tobacco, alcohol, cannabis, and other drugs. Frontiers in Psychiatry. 2021;12.

7. Blithikioti C, Nuño L, Paniello B, Gual A, Miquel L. Impact of COVID-19 lockdown on individuals under treatment for substance use disorders: risk factors for adverse mental health outcomes. Journal of Psychiatric Research. 2021;139:47-53.

8. Bochicchio LA, Drabble LA, Riggle ED, Munroe C, Wootton AR, Hughes TL. Understanding alcohol and marijuana use among sexual minority women during the COVID-19 pandemic: a descriptive phenomenological study. Journal of Homosexuality. 2021;68(4):631-46.

9. Boehnke KF, McAfee J, Ackerman JM, Kruger DJ. Medication and substance use increases among people using cannabis medically during the COVID-19 pandemic. International Journal of Drug Policy. 2021;92:103053.

10. Bonar EE, Chapman L, McAfee J, Goldstick JE, Bauermeister JA, Carter PM, et al. Perceived impacts of the COVID-19 pandemic on cannabis-using emerging adults. Translational Behavioral Medicine. 2021;11(7):1299-309.

11. Bonny-Noach H, Cohen-Louck K, Levy I. Substances use between early and later stages of the COVID-19 pandemic in Israel. Israel Journal of Health Policy Research. 2021;10(1):1-7.

12. Brenneke SG, Nordeck CD, Riehm KE, Schmid I, Tormohlen KN, Smail EJ, et al. Trends in cannabis use among US adults amid the COVID-19 pandemic. International Journal of Drug Policy. 2022;100:103517.

13. Brotto LA, Chankasingh K, Baaske A, Albert A, Booth A, Kaida A, et al. The influence of sex, gender, age, and ethnicity on psychosocial factors and substance use throughout phases of the COVID-19 pandemic. PloS One. 2021;16(11):e0259676.

14. Busse H, Buck C, Stock C, Zeeb H, Pischke CR, Fialho PMM, et al. Engagement in health risk behaviours before and during the COVID-19 pandemic in German university students: results of a cross-sectional study. International Journal of Environmental Research and Public Health. 2021;18(4):1410.

15. Camacho-Rivera M, Islam JY, Rodriguez DL, Vidot DC. Cannabis use among cancer survivors amid the COVID-19 pandemic: Results from the COVID-19 cannabis health study. Cancers. 2021;13(14):3495.

16. Carlyle M, Leung J, Walter ZC, Juckel J, Salom C, Quinn CA, et al. Changes in Substance Use Among People Seeking Alcohol and Other Drug Treatment During the COVID-19 Pandemic: Evaluating Mental Health Outcomes and Resilience. Substance Abuse: Research and Treatment. 2021;15:11782218211061746.

17. Case KR, Clendennen SL, Shah J, Tsevat J, Harrell MB. Changes in marijuana and nicotine vaping perceptions and use behaviors among young adults since the COVID-19 pandemic: A qualitative study. Addictive Behaviors Reports. 2022;15:100408.

18. Chaiton M, Dubray J, Kundu A, Schwartz R. Perceived Impact of COVID on Smoking, Vaping, Alcohol and Cannabis Use Among Youth and Youth Adults in Canada. The Canadian Journal of Psychiatry. 2021:07067437211042132.

19. Clendennen SL, Case KR, Sumbe A, Mantey DS, Mason EJ, Harrell MB. Stress, dependence, and COVID-19–related changes in past 30-day Marijuana, electronic cigarette, and cigarette use among youth and young adults. Tobacco Use Insights. 2021;14:1179173X211067439.

20. Conroy DA, Hadler NL, Cho E, Moreira A, MacKenzie C, Swanson LM, et al. The effects of COVID-19 stay-at-home order on sleep, health, and working patterns: a survey study of US health care workers. Journal of Clinical Sleep Medicine. 2021;17(2):185-91.

21. Cousijn J, Kuhns L, Larsen H, Kroon E. For better or for worse? A pre–post exploration of the impact of the COVID‐19 lockdown on cannabis users. Addiction. 2021;116(8):2104-15.

22. Currie CL. Adult PTSD symptoms and substance use during Wave 1 of the COVID-19 pandemic. Addictive Behaviors Reports. 2021;13:100341.

23. Das A, Singh P, Bruckner TA. State lockdown policies, mental health symptoms, and using substances. Addictive Behaviors. 2022;124:107084.

24. de Quervain D, Aerni A, Amini E, Bentz D, Coynel D, Gerhards C, et al. The Swiss corona stress study. 2020.

25. Dietz P, Werner AM, Reichel JL, Schäfer M, Mülder LM, Beutel M, et al. The Prevalence of Pharmacological Neuroenhancement Among University Students Before and During the COVID-19-Pandemic: Results of Three Consecutive Cross-Sectional Survey Studies in Germany. Frontiers in Public Health. 2022:601.

26. Donovan KA, Portman DG. Effect OF COVID-19 pandemic on cannabis use in cancer patients. American Journal of Hospice and Palliative Medicine®. 2021;38(7):850-3.

27. Dozois DJ. Anxiety and depression in Canada during the COVID-19 pandemic: A national survey. Canadian Psychology. 2021;62(1):136.

28. Dumas TM, Ellis WE, Van Hedger S, Litt DM, MacDonald M. Lockdown, bottoms up? Changes in adolescent substance use across the COVID-19 pandemic. Addictive behaviors. 2022;131:107326.

29. Dyar C, Morgan E, Kaysen D, Newcomb ME, Mustanski B. Risk factors for elevations in substance use and consequences during the COVID-19 pandemic among sexual and gender minorities assigned female at birth. Drug and Alcohol Dependence. 2021;227:109015.

30. El-Gabalawy R, Sommer JL. “We Are at Risk Too”: The Disparate Mental Health Impacts of the Pandemic on Younger Generations: Nous Sommes Aussi à Risque: Les Effets Disparates de la Pandémie Sur la Santé Mentale des Générations Plus Jeunes. The Canadian Journal of Psychiatry. 2021;66(7):634-44.

31. Fedorova EV, Wong CF, Conn BM, Ataiants J, Iverson E, Lankenau SE. COVID-19’s Impact on Substance Use and Well-Being of Younger Adult Cannabis Users in California: A Mixed Methods Inquiry. Journal of Drug Issues. 2021:00220426211052673.

32. Fernández-Artamendi S, Ruiz MJ, López-Núñez C. Analyzing the Behavior of Cannabis Users during the COVID-19 Confinement in Spain. International Journal of Environmental Research and Public Health. 2021;18(21):11324.

33. Firkey MK, Sheinfil AZ, Woolf-King SE. Substance use, sexual behavior, and general well-being of US college students during the COVID-19 pandemic: a brief report. Journal of American College Health. 2020:1-7.

34. Fitzke RE, Wang J, Davis JP, Pedersen ER. Substance use, depression, and loneliness among American veterans during the COVID‐19 pandemic. The American Journal on Addictions. 2021;30(6):552-9.

35. Gaiha SM, Lempert LK, Halpern-Felsher B. Underage youth and young adult e-cigarette use and access before and during the coronavirus disease 2019 pandemic. JAMA network open. 2020;3(12):e2027572-e.

36. Gattamorta KA, Salerno JP, Islam JY, Vidot DC. Mental health among LGBTQ cannabis users during the COVID-19 pandemic: Analysis of the COVID-19 cannabis health study. Psychology of Sexual Orientation and Gender Diversity. 2021;8(2):172.

37. Gaume J, Schmutz E, Daeppen J-B, Zobel F. Evolution of the Illegal Substances Market and Substance Users’ Social Situation and Health during the COVID-19 Pandemic. International Journal of Environmental Research and Public Health. 2021;18(9):4960.

38. Glazer SA, Vallis M. Weight gain, weight management and medical care for individuals living with overweight and obesity during the COVID‐19 pandemic (EPOCH Study). Obesity Science & Practice. 2022.

39. Goodyear T, Slemon A, Richardson C, Gadermann A, Salway T, Dhari S, et al. Increases in Alcohol and Cannabis Use Associated with Deteriorating Mental Health among LGBTQ2+ Adults in the Context of COVID-19: A Repeated Cross-Sectional Study in Canada, 2020–2021. International Journal of Environmental Research and Public Health. 2021;18(22):12155.

40. Graupensperger S, Fleming CB, Jaffe AE, Rhew IC, Patrick ME, Lee CM. Changes in young adults’ alcohol and marijuana use, norms, and motives from before to during the COVID-19 pandemic. Journal of Adolescent Health. 2021;68(4):658-65.

41. Gritsenko V, Skugarevsky O, Konstantinov V, Khamenka N, Marinova T, Reznik A, et al. COVID 19 fear, stress, anxiety, and substance use among Russian and Belarusian university students. International Journal of Mental Health and Addiction. 2021;19(6):2362-8.

42. Hicks TA, Chartier KG, Buckley TD, Reese D, Working Group TSfS, Vassileva J, et al. Divergent changes: abstinence and higher-frequency substance use increase among racial/ethnic minority young adults during the COVID-19 global pandemic. The American Journal of Drug and Alcohol Abuse. 2022;48(1):88-99.

43. Hochstatter KR, Akhtar WZ, Dietz S, Pe-Romashko K, Gustafson DH, Shah DV, et al. Potential influences of the COVID-19 pandemic on drug use and HIV care among people living with HIV and substance use disorders: experience from a pilot mHealth intervention. AIDS and Behavior. 2021;25(2):354-9.

44. Howard K, Grigsby TJ, Haskard-Zolnierek KB, Deason RG, Howard JT. Pandemic-related work status is associated with self-reported increases in substance use. Journal of Workplace Behavioral Health. 2021;36(3):250-7.

45. Imboden C, Claussen MC, Iff S, Quednow BB, Seifritz E, Spörri J, et al. COVID-19 Lockdown 2020 Changed Patterns of Alcohol and Cannabis Use in Swiss Elite Athletes and Bodybuilders: Results From an Online Survey. Frontiers in Sports and Active Living. 2021;3.

46. Imtiaz S, Wells S, Rehm J, Hamilton HA, Nigatu YT, Wickens CM, et al. Cannabis use during the COVID-19 pandemic in Canada: A repeated cross-sectional study. Journal of Addiction Medicine. 2021;15(6):484.

47. Imtiaz S, Wells S, Rehm J, Wickens CM, Hamilton H, Nigatu YT, et al. Daily cannabis use during the novel coronavirus disease (COVID-19) pandemic in Canada: a repeated cross-sectional study from May 2020 to December 2020. Substance Abuse Treatment, Prevention, and Policy. 2022;17(1):1-8.

48. Janulis P, Newcomb ME, Mustanski B. Decrease in prevalence but increase in frequency of non-marijuana drug use following the onset of the COVID-19 pandemic in a large cohort of young men who have sex with men and young transgender women. Drug and Alcohol Dependence. 2021;223:108701.

49. Jodczyk AM, Kasiak PS, Adamczyk N, Gębarowska J, Sikora Z, Gruba G, et al. PaLS Study: Tobacco, Alcohol and Drugs Usage among Polish University Students in the Context of Stress Caused by the COVID-19 Pandemic. International Journal of Environmental Research and Public Health. 2022;19(3):1261.

50. Knell G, Robertson MC, Dooley EE, Burford K, Mendez KS. Health behavior changes during COVID-19 pandemic and subsequent “stay-at-home” orders. International Journal of Environmental Research and Public Health. 2020;17(17):6268.

51. Lake S, Assaf RD, Gorbach PM, Cooper ZD. Selective Changes in Medical Cannabis Use Early in the COVID-19 Pandemic: Findings from a Web-Based Sample of Adults in the United States. Cannabis and Cannabinoid Research. 2022.

52. Lewis N, Sznitman SR. Too Much Information? Excessive Media Use, Maladaptive Coping, and Increases in Problematic Cannabis Use during the COVID-19 Pandemic. Journal of Psychoactive Drugs. 2022:1-10.

53. Lin SL. Generalized anxiety disorder during COVID-19 in Canada: gender-specific association of COVID-19 misinformation exposure, precarious employment, and health behavior change. Journal of Affective Disorders. 2022;302:280-92.

54. Lintzeris N, Deacon RM, Hayes V, Cowan T, Mills L, Parvaresh L, et al. Opioid agonist treatment and patient outcomes during the COVID‐19 pandemic in south east Sydney, Australia. Drug and alcohol review. 2021.

55. Lukács A. Mental well-being of university students in social isolation. European Journal of Health Psychology. 2021.

56. MacEachern KH, Venugopal J, Varin M, Weeks M, Hussain N, Baker MM. Applying a gendered lens to understanding self-reported changes in alcohol and cannabis consumption during the second wave of the COVID-19 pandemic in Canada, September to December 2020. Health Promotion & Chronic Disease Prevention in Canada: Research, Policy & Practice. 2021;41(11).

57. Manthey J, Kilian C, Carr S, Bartak M, Bloomfield K, Braddick F, et al. Use of alcohol, tobacco, cannabis, and other substances during the first wave of the SARS-CoV-2 pandemic in Europe: a survey on 36,000 European substance users. Substance Abuse Treatment, Prevention, and Policy. 2021;16(1):1-11.

58. Meanley S, Choi SK, Thompson AB, Meyers JL, D’Souza G, Adimora AA, et al. Short-term binge drinking, marijuana, and recreational drug use trajectories in a prospective cohort of people living with HIV at the start of COVID-19 mitigation efforts in the United States. Drug and Alcohol Dependence. 2022;231:109233.

59. Merrill JE, Stevens AK, Jackson KM, White HR. Changes in cannabis consumption among college students during CoViD-19. Journal of Studies on Alcohol and Drugs. 2022;83(1):55-63.

60. Mezaache S, Donadille C, Martin V, Le Brun Gadelius M, Appel L, Spire B, et al. Changes in cannabis use and associated correlates during France’s first COVID-19 lockdown in daily cannabis users: results from a large community-based online survey. Harm Reduction Journal. 2022;19(1):1-10.

61. Miech R, Patrick ME, Keyes K, O’Malley PM, Johnston L. Adolescent drug use before and during US national COVID-19 social distancing policies. Drug and alcohol dependence. 2021;226:108822.

62. Miller K, Laha-Walsh K, Albright DL, McDaniel J. Cannabis use during the COVID-19 pandemic: results from a longitudinal study of Cannabis users. Journal of Substance Use. 2022;27(1):38-42.

63. Mohr CD, Umemoto SK, Rounds TW, Bouleh P, Arpin SN. Drinking to cope in the COVID-19 era: an investigation among college students. Journal of Studies on Alcohol and Drugs. 2021;82(2):178-87.

64. Mora AM, Lewnard JA, Kogut K, Rauch S, Morga N, Jewell N, et al. Impact of the COVID-19 pandemic and vaccine hesitancy among farmworkers from Monterey County, California. medRxiv. 2020.

65. Mravčík V, Chomynová P. Substance use and addictive behaviours during COVID-19 confinement measures increased in intensive users: Results of an online general population survey in the Czech Republic. Epidemiologie, mikrobiologie, imunologie: casopis Spolecnosti pro epidemiologii a mikrobiologii Ceske lekarske spolecnosti JE Purkyne. 2021;70(2):98-103.

66. Nguyen N, Gaiha SM, Halpern-Felsher B. Self-reported changes in cannabis vaping among US adolescents and young adults early in the COVID-19 pandemic. Preventive Medicine Reports. 2021;24:101654.

67. Otiashvili D, Mgebrishvili T, Beselia A, Vardanashvili I, Dumchev K, Kiriazova T, et al. The impact of the COVID-19 pandemic on illicit drug supply, drug-related behaviour of people who use drugs and provision of drug related services in Georgia: results of a mixed methods prospective cohort study. Harm Reduction Journal. 2022;19(1):1-15.

68. Palamar JJ, Le A, Acosta P. Shifts in drug use behavior among electronic dance music partygoers in New York during COVID-19 social distancing. Substance Use & Misuse. 2020;56(2):238-44.

69. Papp LM, Kouros CD. Effect of COVID-19 disruptions on young adults’ affect and substance use in daily life. Psychology of Addictive Behaviors. 2021.

70. Pavarin RM, Bettelli S, Nostrani E, Mazzotta C, Salsano V, Ulgheri AL, et al. Substance consumption styles during the COVID-19 lockdown for socially integrated people who use drugs. Journal of Substance Use. 2022;27(2):218-23.

71. Pedersen ER, Davis JP, Fitzke RE, Lee DS, Saba S. American Veterans in the Era of COVID-19: Reactions to the Pandemic, Posttraumatic Stress Disorder, and Substance Use Behaviors. International Journal of Mental Health and Addiction. 2021:1-16.

72. Pocuca N, London-Nadeau K, Geoffroy M-C, Chadi N, Séguin JR, Parent S, et al. Changes in emerging adults’ alcohol and cannabis use from before to during the COVID-19 pandemic: Evidence from a prospective birth cohort. Psychology of Addictive Behaviors. 2022.

73. Potvin J, Ramos Socarras L, Forest G. Sleeping through a lockdown: How adolescents and young adults struggle with lifestyle and sleep habits upheaval during a pandemic. Behavioral Sleep Medicine. 2022:1-17.

74. Rantis K, Panagiotidis P, Parlapani E, Holeva V, Tsapakis E, Diakogiannis I. Substance use during the COVID-19 pandemic in Greece. Journal of Substance Use. 2021:1-8.

75. Reilly ED, Chamberlin ES, Duarte BA, Harris JI, Shirk SD, Kelly MM. The Impact of COVID-19 on Self-Reported Substance Use, Well-Being, and Functioning Among United States Veterans: A Cross-Sectional Study. Frontiers in Psychology. 2022;13:812247.

76. Reuter PR, Forster BL, Kruger BJ. A longitudinal study of the impact of COVID-19 restrictions on students’ health behavior, mental health and emotional well-being. PeerJ. 2021;9:e12528.

77. Robillard R, Daros AR, Phillips JL, Porteous M, Saad M, Pennestri M-H, et al. Emerging New Psychiatric Symptoms and the Worsening of Pre-existing Mental Disorders during the COVID-19 Pandemic: A Canadian Multisite Study: Nouveaux symptômes psychiatriques émergents et détérioration des troubles mentaux préexistants durant la pandémie de la COVID-19: une étude canadienne multisite. The Canadian Journal of Psychiatry. 2021;66(9):815-26.

78. Rodriguez DL, Vidot DC, Camacho-Rivera M, Islam JY. Mental health symptoms during the COVID-19 pandemic among cancer survivors who endorse cannabis: Results from the COVID-19 cannabis health study. Current Oncology. 2022;29(3):2106-18.

79. Rogers AH, Shepherd JM, Garey L, Zvolensky MJ. Psychological factors associated with substance use initiation during the COVID-19 pandemic. Psychiatry Research. 2020;293:113407.

80. Rolland B, Haesebaert F, Zante E, Benyamina A, Haesebaert J, Franck N. Global changes and factors of increase in caloric/salty food intake, screen use, and substance use during the early COVID-19 containment phase in the general population in France: survey study. JMIR Public Health and Surveillance. 2020;6(3):e19630.

81. Romm KF, Patterson B, Crawford ND, Posner H, West CD, Wedding D, et al. Changes in young adult substance use during COVID-19 as a function of ACEs, depression, prior substance use and resilience. Substance Abuse. 2022;43(1):212-21.

82. Romm KF, Patterson B, Arem H, Price OA, Wang Y, Berg CJ. Cross-sectional retrospective assessments versus longitudinal prospective assessments of substance use change among young adults during COVID-19: magnitude and correlates of discordant findings. Substance Use & Misuse. 2022;57(3):484-9.

83. Salles J, Yrondi A, Marhar F, Andant N, Dorlhiac RA, Quach B, et al. Changes in cannabis consumption during the global covid-19 lockdown: The international COVISTRESS study. Frontiers in Psychiatry. 2021:1985.

84. Salmon S, Taillieu TL, Fortier J, Stewart-Tufescu A, Afifi TO. Pandemic-related experiences, mental health symptoms, substance use, and relationship conflict among older adolescents and young adults from Manitoba, Canada. Psychiatry Research. 2022;311:114495.

85. Sande M, Šabić S, Paš M, Verdenik M. How has the COVID-19 Epidemic Changed Drug Use and the Drug Market in Slovenia? Društvena istraživanja. 2021;30(2):313-32.

86. Schepis TS, De Nadai AS, Bravo AJ, Looby A, Villarosa-Hurlocker MC, Earleywine M, et al. Alcohol use, cannabis use, and psychopathology symptoms among college students before and after COVID-19. Journal of Psychiatric Research. 2021;142:73-9.

87. Scherbaum N, Bonnet U, Hafermann H, Schifano F, Bender S, Grigoleit T, et al. Availability of illegal drugs during the CoViD-19 pandemic in western Germany. Frontiers in psychiatry. 2021;12.

88. Shapiro O, Gannot RN, Green G, Zigdon A, Zwilling M, Giladi A, et al. Risk Behaviors, Family Support, and Emotional Health among Adolescents during the COVID-19 Pandemic in Israel. International Journal of Environmental Research and Public Health. 2022;19(7):3850.

89. Sharma P, Ebbert JO, Rosedahl JK, Philpot LM. Changes in substance use among young adults during a respiratory disease pandemic. SAGE Open Medicine. 2020;8:2050312120965321.

90. Slemon A, Richardson C, Goodyear T, Salway T, Gadermann A, Oliffe JL, et al. Widening mental health and substance use inequities among sexual and gender minority populations: Findings from a repeated cross-sectional monitoring survey during the COVID-19 pandemic in Canada. Psychiatry research. 2022;307:114327.

91. Somé NH, Wells S, Felsky D, Hamilton HA, Ali S, Elton-Marshall T, et al. Self-reported mental health during the COVID-19 pandemic and its association with alcohol and cannabis use: a latent class analysis. BMC Psychiatry. 2022;22(1):1-13.

92. Somé NH, Shokoohi M, Shield KD, Wells S, Hamilton HA, Elton-Marshall T, et al. Alcohol and cannabis use during the COVID-19 pandemic among transgender, gender-diverse, and cisgender adults in Canada. BMC Public Health. 2022;22(1):1-9.

93. Starks TJ, Jones SS, Sauermilch D, Benedict M, Adebayo T, Cain D, et al. Evaluating the impact of COVID-19: A cohort comparison study of drug use and risky sexual behavior among sexual minority men in the USA. Drug and Alcohol Dependence. 2020;216:108260.

94. Studer J, Marmet S, Gmel G, Wicki M, Labhart F, Gachoud C, et al. Changes in substance use and other reinforcing behaviours during the COVID-19 crisis in a general population cohort study of young Swiss men. Journal of Behavioral Addictions. 2021.

95. Sylvestre M-P, Dinkou GDT, Naja M, Riglea T, Pelekanakis A, Bélanger M, et al. A longitudinal study of change in substance use from before to during the COVID-19 pandemic in young adults. The Lancet Regional Health-Americas. 2022;8:100168.

96. Sznitman S, Rosenberg D, Lewis N. Are COVID-19 health-related and socioeconomic stressors associated with increases in cannabis use in individuals who use cannabis for recreational purposes? Substance Abuse. 2022;43(1):301-8.

97. Tavolacci MP, Wouters E, Van de Velde S, Buffel V, Déchelotte P, Van Hal G, et al. The impact of COVID-19 lockdown on health behaviors among students of a French university. International Journal of Environmental Research and Public Health. 2021;18(8):4346.

98. Terry-McElrath YM, O’Malley PM, Pang YC, Patrick ME. Characteristics and reasons for use associated with solitary alcohol and marijuana use among US 12th Grade Students, 2015–2021. Drug and Alcohol Dependence. 2022;235:109448.

99. Tham SW, Murray CB, Law EF, Slack KE, Palermo TM. The impact of the coronavirus disease 2019 pandemic on pain and psychological functioning in young adults with chronic pain. Pain. 2022:10.1097.

100. Tholen R, Ponnet K, Van Hal G, De Bruyn S, Buffel V, Van de Velde S, et al. Substance use among Belgian higher education students before and during the first wave of the COVID-19 pandemic. International Journal of Environmental Research and Public Health. 2022;19(7):4348.

101. Tucker JS, D'Amico EJ, Pedersen ER, Garvey R, Rodriguez A, Klein DJ. Behavioral health and service usage during the COVID-19 pandemic among emerging adults currently or recently experiencing homelessness. Journal of Adolescent Health. 2020;67(4):603-5.

102. Turna J, Patterson B, Goldman Bergmann C, Lamberti N, Rahat M, Dwyer H, et al. Mental health during the first wave of COVID-19 in Canada, the USA, Brazil and Italy. International Journal of Psychiatry in Clinical Practice. 2021:1-9.

103. Vanderbruggen N, Matthys F, Van Laere S, Zeeuws D, Santermans L, Van den Ameele S, et al. Self-reported alcohol, tobacco, and cannabis use during COVID-19 lockdown measures: results from a web-based survey. European Addiction Research. 2020;26(6):309-15.

104. van Hooijdonk KJ, Rubio M, Simons SS, van Noorden TH, Luijten M, Geurts SA, et al. Student-, Study-and COVID-19-Related Predictors of Students’ Smoking, Binge Drinking and Cannabis Use before and during the Initial COVID-19 Lockdown in The Netherlands. International Journal of Environmental Research and Public Health. 2022;19(2):812.

105. Van Laar MW, Oomen PE, Van Miltenburg CJ, Vercoulen E, Freeman TP, Hall WD. Cannabis and COVID-19: reasons for concern. Frontiers in Psychiatry. 2020:1419.

106. Varin M, MacEachern KH, Hussain N, Baker MM. At-a-glance-Measuring self-reported change in alcohol and cannabis consumption during the second wave of the COVID-19 pandemic in Canada. Health Promotion and Chronic Disease Prevention in Canada: Research, Policy and Practice. 2021;41(11):325.

107. Vedelago L, Wardell J, Kempe T, Patel H, Amlung M, MacKillop J, et al. Getting high to cope with COVID-19: Modelling the associations between cannabis demand, coping motives, and cannabis use and problems. Addictive Behaviors. 2022;124:107092.

108. Vidot DC, Islam JY, Camacho-Rivera M, Harrell MB, Rao DR, Chavez JV, et al. The COVID-19 cannabis health study: Results from an epidemiologic assessment of adults who use cannabis for medicinal reasons in the United States. Journal of Addictive Diseases. 2020;39(1):26-36.

109. Villanti AC, LePine SE, Peasley‐Miklus C, West JC, Roemhildt M, Williams R, et al. COVID‐related distress, mental health, and substance use in adolescents and young adults. Child and Adolescent Mental Health. 2022;27(2):138-45.

110. von Soest T, Kozák M, Rodríguez-Cano R, Fluit DH, Cortés-García L, Ulset VS, et al. Adolescents’ psychosocial well-being one year after the outbreak of the COVID-19 pandemic in Norway. Nature Human Behaviour. 2022;6(2):217-28.

111. Wang Y, Ibañez GE, Vaddiparti K, Stetten NE, Sajdeya R, Porges EC, et al. Change in marijuana use and its associated factors among persons living with HIV (PLWH) during the COVID-19 pandemic: findings from a prospective cohort. Drug and Alcohol Dependence. 2021;225:108770.

112. Weber CAT, Monteiro IT, Gehrke JM, de Souza WS. The Use of Psychoactive Substances in the Context of the Covid-19 Pandemic in Brazil. medRxiv. 2020.

113. Weyandt LL, Francis A, Shepard E, Gudmundsdóttir BG, Channell I, Beatty A, et al. Anxiety, depression, impulsivity, and mindfulness among higher education faculty during covid-19. Health Behavior and Policy Review. 2020;7(6):532-45.

114. Xuereb S, Kim HS, Clark L, Wohl MJ. Substitution behaviors among people who gamble during COVID-19 precipitated casino closures. International Gambling Studies. 2021;21(3):411-25.

115. Yousufzai SJ, Cole AG, Nonoyama M, Barakat C. Changes in cannabis consumption among emerging adults in relation to policy and public health developments. Substance Use & Misuse. 2022;57(5):730-41.

116. Zajacova A, Jehn A, Stackhouse M, Denice P, Ramos H. Changes in health behaviours during early COVID-19 and socio-demographic disparities: a cross-sectional analysis. Canadian Journal of Public Health. 2020;111(6):953-62.

117. Doherty L, Sullivan, T., & Voce, A. . Impact of the COVID-19 pandemic on cannabis demand and supply in Australia. 2021.

118. Peacock A, Karlsson A, Uporova J, Price O, Chan R, Swanton R, et al. Australian Drug Trends 2020: Key Findings from the National Ecstasy and Related Drugs Reporting System (EDRS) Interviews. . 2020.

119. Bruton L, Featherstone T, Gibney S. Impact of COVID-19 on Drug and Alcohol Services and People who use Drugs in Ireland: A report of survey findings.; 2021.

120. Johnston LD, Miech RA, O'Malley PM, Bachman JG, Schulenberg JE, Patrick ME. Monitoring the Future national survey results on drug use, 1975-2021: Overview, key findings on adolescent drug use. 2022.

121. Aldridge J, Garius L, Spicer J, Harris M, Moore K, Eastwood N. Drugs in the time of COVID: the UK drug market response to lockdown restrictions. 2021.

122. Statistics Canada. Alcohol and cannabis use during the pandemic: Canadian Perspectives Survey Series 6.; 2021.

123. NANOS Research. COVID-19 and increased alcohol consumption: NANOS poll summary report. Canadian Centre on Substance Use and Addiction Ottawa ON, Canada; 2020.

124. Baillie G, Peacock A, Hammoud M, Memedovic S, Barratt M, Bruno R, et al. Key findings from the ‘Australians’ Drug Use: Adapting to Pandemic Threats (ADAPT)’Study Wave 4. ADAPT Bulletin. 2021(4).

125. EMCDDA. The European Monitoring Centre for Drugs and Drug Addiction (EMCDDA) European Web Survey on Drugs: Results.; 2022.

126. Peacock A, Uporova J, Karlsson A, Price O, Gibbs D, Swanton R, et al. Australian drug trends 2020: key findings from the National Illicit Drug Reporting System (IDRS) interviews. 2021.

127. Mental Health Commission of Canada. Mental Health and Substance Use During COVID‐19: Summary Report. 2021.

128. New Zealand Drug Foundation. Pulse survey during Alert Level Four of addiction services and people who use drugs in New Zealand.; 2020.

129. Statistics Canada. Table 13-10-0383-01  Prevalence of cannabis use in the past three months, self-reported. 2020.
